# PlantEAR: Functional Analysis Platform for Plant EAR Motif-Containing Proteins

**DOI:** 10.3389/fgene.2018.00590

**Published:** 2018-11-30

**Authors:** Jiaotong Yang, Yue Liu, Hengyu Yan, Tian Tian, Qi You, Liwei Zhang, Wenying Xu, Zhen Su

**Affiliations:** State Key Laboratory of Plant Physiology and Biochemistry, College of Biological Sciences, China Agricultural University, Beijing, China

**Keywords:** EAR motif, transcription repression, functional annotations, network analysis, functional enrichment analysis

## Abstract

The Ethylene-responsive element binding factor-associated Amphiphilic Repression (EAR) motifs, which were initially identified in members of the *Arabidopsis* ethylene response factor (ERF) family, are transcriptional repression motifs in plants and are defined by the consensus sequence patterns of either LxLxL or DLNxxP. EAR motif-containing proteins can function as transcription repressors, thus interacting with co-repressors, such as TOPLESS and AtSAP18, affecting the structure of chromatin by histone modifications and thereby repressing gene transcription. EAR motif-containing proteins are highly conserved across diverse plant species and play important roles in hormone signal transduction, stress responses and development, but they have not been identified in most plants. In this study, we identified 20,542 EAR motif-containing proteins from 71 plant species based on a Hidden Markov Model and orthologous gene search, and then we constructed a functional analysis platform for plant EAR motif-containing proteins (PlantEAR, http://structuralbiology.cau.edu.cn/plantEAR) by integrating a variety of functional annotations and processed data. Several tools were provided as functional support for EAR motif-containing proteins, such as browse, search, co-expression and protein-protein interaction (PPI) network analysis as well as cis-element analysis and gene set enrichment analysis (GSEA). In addition, basing on the identified EAR motif-containing proteins, we also explored their distribution in various species and found that the numbers of EAR motif-containing proteins showed an increasing trend in evolution from algae to angiosperms.

## Introduction

EAR motifs, which were initially identified in members of the *Arabidopsis* ethylene response factor (ERF) family, are emerging as transcriptional repression motifs in plants (Ohta et al., 2001). EAR motif-mediated transcriptional repression is the main form of transcriptional repression identified in plants thus far (Kagale and Rozwadowski, 2011; Causier et al., 2012; Ma et al., 2017; Wu and Citovsky, 2017; Choi et al., 2018). There are two separate ways for EAR motif-containing proteins to regulate gene transcription (Kagale and Rozwadowski, 2011). First, EAR motif-containing proteins can serve as transcription factors (TFs), thus binding to the promoter region of genes, affecting chromatin structure by histone modifications, and thereby repressing gene transcription. EAR motif-containing proteins can recruit a histone deacetylase (HDAC) complex to perform transcriptional repression functions by interacting with co-suppressors, such as *SAP18* and *TOPLESS*, in *Arabidopsis thaliana* (Song et al., 2005; Song and Galbraith, 2006; Hill et al., 2008; Causier et al., 2012). Several members of the ERF family contain the DLNxxP type EAR motif and play important roles in active repression. Obvious examples are *AtERF3* and *AtERF4*, which can be bound by *AtSAP18* and recruit *AtHDA19* to form the HDAC complex to repress gene expression (Fujimoto et al., 2000; McGrath et al., 2005; Yang et al., 2005). *AtERF7* can also interact with *AtSIN3* to recruit *AtHDA19* to form the HDAC complex and repress gene transcription (Song et al., 2005). Second, EAR motif-containing proteins can function as transcription regulators (TRs) regulating the functional status of the transcriptional activators by directly or indirectly binding to them. Obvious examples are JASMONATE ZIM domain (JAZ) proteins with EAR motifs in *Arabidopsis thaliana*, which can negatively regulate the key transcriptional activators of jasmonate responses, such as *MYC2, MYC3, MYC4, MYC5* (Chini et al., 2007; Kagale et al., 2010; Goossens et al., 2015; Thireault et al., 2015; Song et al., 2017).

EAR motif-containing proteins have many important functions in different plant species. (1) EAR motif-containing proteins are responsible for the regulation of the phytohormone signaling pathway. In *Arabidopsis thaliana*, jasmonate ZIM domain (JAZ), and indole-3-acetic acid (auxin, IAA) proteins are important signaling molecules of the jasmonic acid (JA) and indole-3-acetic acid (auxin, IAA) pathway, respectively, and many of them are EAR motif-containing proteins (Chini et al., 2007; Guilfoyle and Hagen, 2007; Kagale et al., 2010). *DWARF 53* (*D53*) can work as a suppressor of strigolactone (SL) signaling in rice (Jiang et al., 2013), and its orthologous protein *SMAX1-LIKE 7* (*SMXL7*) in *Arabidopsis thaliana* can also function in the response process of SL (Liang et al., 2016). (2) Several EAR motif-containing proteins have been described in the regulation of plant growth and development. The EAR motif-containing protein *BRASSINAZOLE-RESISTANT 2* (*BES1, BZR2*) is involved in the regulation of organ boundary formation in the shoot apical meristem and cell division in the root quiescent center (QC; Espinosa-Ruiz et al., 2017). For leaf development, the EAR motif-containing proteins *KIX8* and *KIX9* can work as adaptor proteins for the corepressor *TOPLESS* to repress leaf growth (Gonzalez et al., 2015). For flower development, *TOE1*, and *TOE2* have been reported to repress expression of *FLOWERING LOCUS T* (*FT*) by interacting with *TOPLESS*, thereby repressing flowering in *Arabidopsis thaliana* (Zhai et al., 2015). (3) EAR motif-containing proteins have an important effect on the stress response. Several ERFs containing an EAR motif function as transcriptional repressors in many species and are known to play roles in wounding, cold, salt and drought stress responses, such as *AtERF4* (Ohta et al., 2001; McGrath et al., 2005), *AtERF7* (Song et al., 2005), *NtERF3* (Ohta et al., 2001), *SodERF3* (Trujillo et al., 2008), *GhERF4* (Jin and Liu, 2008), *OsERF3* (Lu et al., 2011), *OsBIERF2* (Cao et al., 2006), *GmERF4* (Zhang et al., 2010), *TaERF4* (Dong et al., 2012), *SiERF3* (Pan et al., 2010).

A rapidly accumulating sequence of plant species are available from many public platforms, such as Phytozome (Goodstein et al., 2012) and PlantGDB (Duvick et al., 2008). Several platforms have been established to store and analyse specific factors, such as the plant transcription factor database (PlantTFDB; Jin et al., 2017) and leaf senescence database (LSD; Li et al., 2014), which can effectively advance the study of plant transcriptional regulation and leaf senescence processes. The EAR motif-containing proteins are a class of proteins that are specifically involved in plant developmental and physiological processes by transcriptional repression (Kagale and Rozwadowski, 2011), so we constructed a platform for plant EAR motif-containing proteins named PlantEAR based on the predicted EAR motif-containing proteins in different species. We also provide several analysis tools, such as function search, browse, network analysis, cis-element analysis, and gene set enrichment analysis (GSEA). In addition, we performed a preliminary phylogenetic analysis of the EAR motif-containing proteins across species. We hope our PlantEAR platform will promote research on the relationship between transcriptional repression and epigenetic modification in the plant community.

## Materials and methods

### Data sources

The data source of this study is presented in Supplementary Table 1. The basic data for the EAR motif-containing proteins in plants was collected from the Phytozome (Goodstein et al., 2012), The Plant Genome Integrative Explorer Resource (PlantGenIE; Sundell et al., 2015), The *Arabidopsis thaliana* Information Resource (TAIR; Swarbreck et al., 2008), Sol Genomics Network (SGN; Fernandez-Pozo et al., 2015), GIGAdb (Guan et al., 2016), ccNET (You et al., 2017), and DRYAD database, including gene annotation information, gene location, transcript sequences, CDS sequences, and protein sequences. KEGG and GO annotation were obtained from the KEGG (Wixon and Kell, 2000) and amiGO database (Carbon et al., 2009), respectively. For *Arabidopsis thaliana*, the phenotypes of EAR motif-containing protein coding genes were collected from *Arabidopsis* Hormone Database 2.0 (AHB2.0; Jiang et al., 2011), and microarray expression profiling of abiotic stress, biotic stress, hormone response, growth, and development (Supplementary Table 2) were collected from the Gene Expression Omnibus (GEO; Clough and Barrett, 2016) in NCBI. The gene co-expression network of *Arabidopsis thaliana, Sorghum bicolor, Oryza sativa, Triticum aestivum, Glycine max, Zea mays* and *Solanum lycopersicum* were obtained from ATTED-II (Aoki et al., 2016), SorghumFDB (Tian et al., 2016), RiceNET (Lee et al., 2015), wheatNET (Lee et al., 2017), soyNET (Kim E. et al., 2017), MCENet (Tian et al., 2018), and TomatoNET (Kim H. et al., 2017), respectively. The co-expression network of *Gossypium arboreum and Gossypium hirsutum* was obtained from ccNET (You et al., 2017). Protein-protein interaction (PPI) networks of *Arabidopsis thaliana* were obtained from the BioGRID (Chatr-Aryamontri et al., 2017), TAIR (Swarbreck et al., 2008), BAR (Waese and Provart, 2017), and CCSB databases (Arabidopsis Interactome Mapping, 2011). Epigenetic modifications for *Arabidopsis thaliana, Oryza sativa* and *Zea mays* were collected from the PCSD (Liu et al., 2018). Cis-elements (motifs) in gene promoters were collected from ccNET, which collected cis-elements from the PLACE database (Higo et al., 1998), PlantCARE database (Rombauts et al., 1999), AthaMap web (Hehl and Bulow, 2014), and reported literature (Chen et al., 2002; Teakle et al., 2002; Bao et al., 2004; Zhang et al., 2012).

### Identification of EAR motif-containing proteins

**Figure 1:**
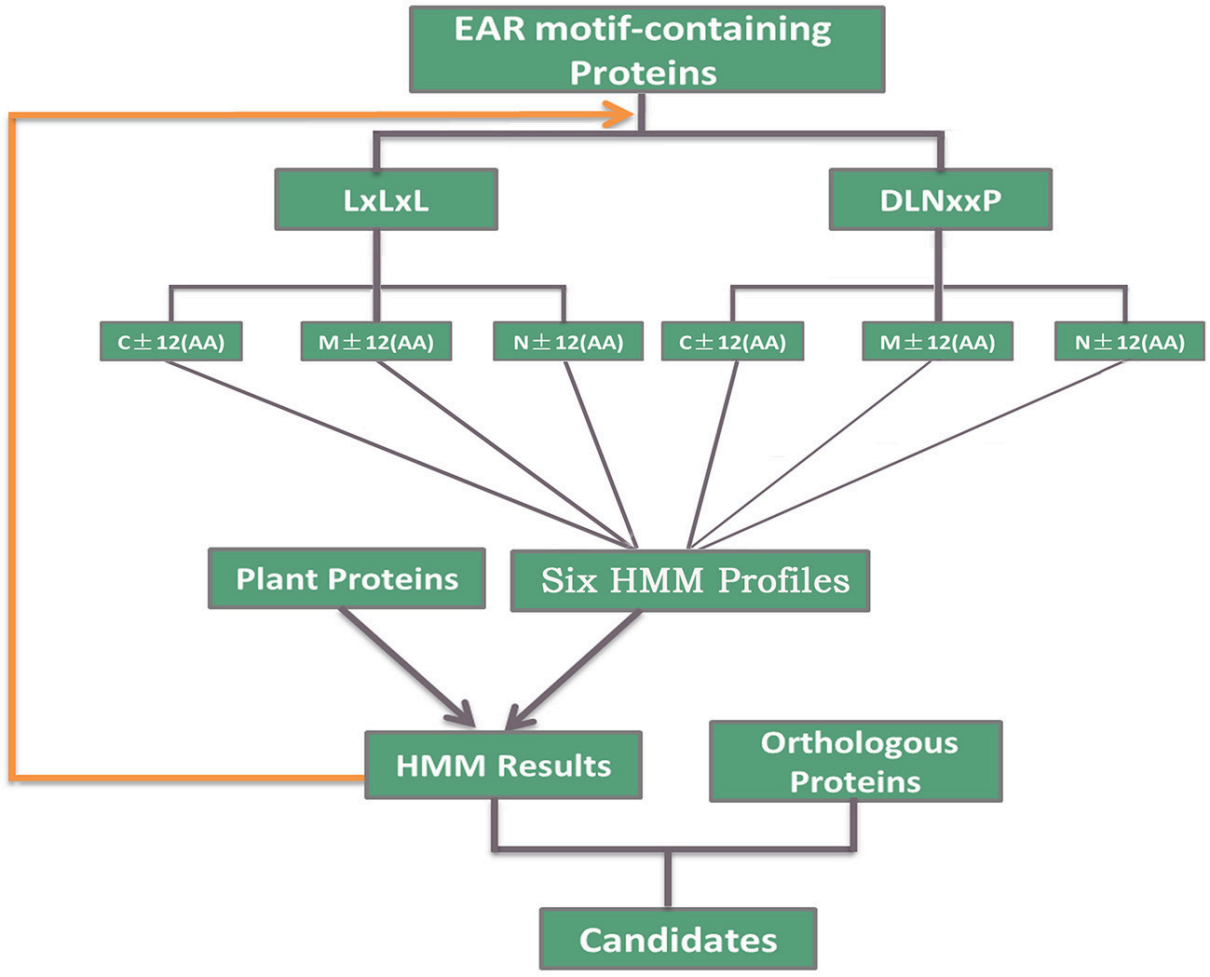
Description of the identification of EAR motif-containing proteins. A total of 219 high-confidence EAR motif-containing proteins in *Arabidopsis* were divided into six subgroups according to the EAR motif pattern (LxLxL, DLNxxP) and the location of the EAR motif (C-terminal, Middle, N-terminal). Iterated HMM was used to identify the EAR motif-containing proteins that would later be filtered with the EAR pattern in each iteration. Inparanoid software was also used to identify the orthologous proteins of 398 *Arabidopsis* EAR motif-containing proteins. Then, we took both HMM and inparanoid tool results with the EAR pattern into consideration and defined the EAR motif-containing protein candidates in plants.

A total of 398 EAR motif-containing proteins were collected in *Arabidopsis thaliana*, including 219 high-confidence EAR motif-containing proteins and another 179 candidates (Kagale et al., 2010). For the identification of putative EAR motif-containing proteins in plants, HMMER (Eddy, 1998) and the inparanoid tool (Sonnhammer and Ostlund, 2015) were used to predict the EAR motif-containing proteins in plants. First, six hidden Markov model (HMM) profiles were constructed using the EAR motif and its adjoining 12-amino-acid sequence of 219 EAR motif-containing proteins based on the EAR motif locations (C-terminal, Middle, N-terminal) and patterns [LxLxL, DLNx[1,2]P; Figure 1]. Second, Each HMM profile was used to evaluate the appearance of novel EAR motif-containing proteins in the proteome of different plants via the hmmsearch command with an e-value < 0.01. Third, we used the hmmsearch results [containing LxLxL or DLNx[1,2]P] and 219 EAR motif-containing proteins to re-construct the HMM profiles as above. Subsequent iterations of hmmsearch and HMM profile re-construction were performed until no more novel EAR motif-containing proteins were presented. Third, based on the protein sequences in *Arabidopsis thaliana*, orthologous genes of other plants were predicted by the inparanoid tool (Sonnhammer and Ostlund, 2015). Then, the orthologous genes of 398 EAR motif-containing proteins in *Arabidopsis thaliana* were added to the EAR motif-containing protein candidates. Finally, combining the hmmsearch with the inparanoid results [containing LxLxL or DLNx[1,2]P] led to the EAR motif-containing protein candidates in different plants (Figure 1).

### Prediction of orthologous proteins

Inparanoid (Sonnhammer and Ostlund, 2015) is a wide used program for predicting orthologous proteins. This program requires BLAST software during its operation. We chose a cutoff of over 60% bootstrap to produce orthologs between *Arabidopsis thaliana* and other species, which facilitated the acquisition of orthologous EAR motif-containing proteins pairs.

### Protein-protein interaction and co-expression network

The PPI networks of *Gossypium arboreum* and *Gossypium hirsutum* were obtained from ccNET, which is predicted by using orthologous relationships. By using the same method, we predicted the PPI network in many other species.

We obtained co-expression networks of 9 species from public platforms (Supplementary Table 1). The co-expression network of *Oryza sativa* (Lee et al., 2015), *Triticum aestivum* (Lee et al., 2017), *Glycine max* (Kim E. et al., 2017) and *Solanum lycopersicum* (Kim H. et al., 2017) was determined by calculating the Pearson correlation coefficient (PCC) between genes. The co-expression networks of *Arabidopsis thaliana* (Aoki et al., 2016), *Zea mays* (Tian et al., 2018), *Sorghum bicolor* (Tian et al., 2016)*, Gossypium arboretum*, and *Gossypium hirsutum* (You et al., 2017) were determined by calculating the Pearson correlation Coefficient (PCC) and mutual rank (MR) between genes.

### Functional module prediction

CFinder (version 2.0.6; Niklas et al., 2015) was used to find group nodes connected more densely than nodes outside the group in gene networks, and these nodes connected densely constitute the functional modules. The parameter k indicated that each node had co-expression interactions with k-1 nodes in a module. Here, we used CFinder results when k = 4 as functional modules for the *Arabidopsis* co-expression network.

### Microarray analysis

The Affymetrix microarray series matrix files were collected from GEO, which is preprocessed by Affymetrix Microarray Suite 5.0 (MAS5), including background correction, normalization, and calculation of expression values. For microarray expression profiles of abiotic stress and hormonal response in *Arabidopsis* (Supplementary Table 2), differential expressed EAR motif-containing protein-coding genes at each time point were calculated using student's *t*-test (*p* < 0.05) and fold change [|log2(foldchange)| > 1].

### Gene family classification

The iTAK software (Zheng et al., 2016) was used to identify transcription factors, transcription regulators and protein kinases, which can be downloaded from the iTAK website (http://itak.feilab.net/cgi-bin/itak/index.cgi; Zheng et al., 2016) and installed locally. We used locally installed iTAK software (Zheng et al., 2016) to identify the gene families of plant proteins. Then, we mapped the predicted classification of gene families to the identified EAR motif-containing proteins. To determine the functional domains, locally installed Pfamscan software (http://pfam.xfam.org/; Finn et al., 2016) was used to predict domains of the plant EAR motif-containing proteins.

### Gene set enrichment analysis

Gene set enrichment analysis (GSEA; Yi et al., 2013) is an online web-sever for gene sets annotation by calculating the overlaps with various well-defined gene sets. Additionally, statistically enriched gene sets were defined by the hypergeometric test, Fisher's exact test or χ2 test. Gene set enrichment analysis tool for multiple species with default parameters (Fisher's exact test, *P* ≤ 0.05) is provided for functional enrichment analysis in plantEAR.

### Cis-element enrichment analysis

Cis-element (motifs), which are a series of short conserved sequences, can be recognized by several transcription factors and then involved in the regulation of downstream genes. Motifs were collected from ccNET (http://structuralbiology.cau.edu.cn/gossypium/) in regular expression format. For “n” genes submitted by users, the Z score and *P*-value of each motif is calculated using the following method. Firstly, we scanned the 3-kb promoter region of these “n” genes and defined the number ($\overset{-}{X}$) of matches to each motif, which represented the number of this motif presented in the 3-kb promoter region of these “n” genes. Secondly, 1,000 random lists with exact “n” genes are obtained, and the 3-kb promoter region of each random list are scanned, and the average number (μ) of each motif are counted. Finally, the Z score and *P*-value for each motif are calculated by the following equations. Those motifs with a *P* < 0.05 were significantly enriched for the “n” genes users submitted (Yu et al., 2014; You et al., 2015).

$$\begin{array}{l} Z=\frac{\bar{X}-\mu }{\sigma /\sqrt{n}}\\ p-value=pnorm\left(\bar{X},\mu ,\frac{\sigma }{\sqrt{n}}\right) \end{array}$$

$\bar{X}$, number of matches to a motif in the 3-kb promoter region of a list of genes; μ, average number of matches to the same motif in the 3-kb promoter region of 1,000 random lists of genes; σ, standard deviation of numbers of the motif to 1,000 random selection; n, number of genes. Pnorm is the distribution function for the normal distribution in the R package.

### Phylogenetic analysis

In plantEAR, we performed a phylogenetic analysis for the same gene family of EAR motif-containing proteins between a model plant *Arabidopsis* and one other plant. In this process, we used Muscle software (Edgar, 2004) to perform the multiple sequence alignments, and the R language ape package (Paradis et al., 2004) was used to construct and visualize the phylogenetic trees. For the phylogenetic analysis of the multi-species EAR motif-containing proteins gene family, Mega6 (Tamura et al., 2013) was used for multiple sequence alignments and phylogenetic tree construction. Phylogenetic trees were constructed using the maximum likelihood method with 1,000 bootstrap replicates. EvolView (He et al., 2016) was then used to show the phylogenetic trees.

## Construction

### Functional annotations

**Figure 2:**
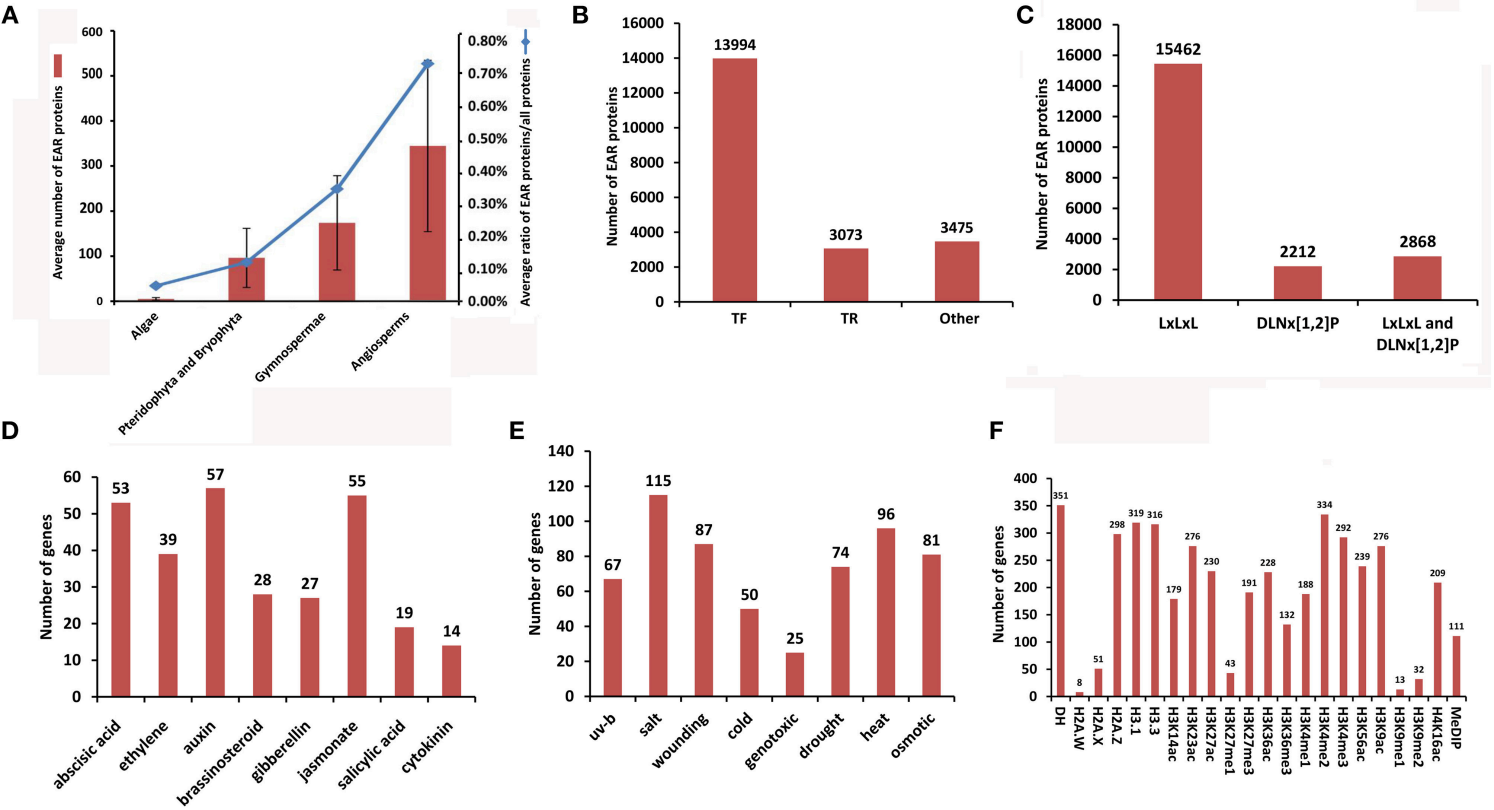
Summary of the statistics for EAR motif-containing proteins in plantEAR. **(A)** Number of EAR motif-containing proteins and the ratio of EAR motif-containing proteins to plant total proteins at different stages of evolution. **(B)** Number of transcription factors (TFs), transcriptional regulators (TRs) and other proteins with EAR motifs in plantEAR. **(C)** Number of EAR motif-containing proteins with different EAR patterns. **(D)** Number of genes encoding EAR motif-containing proteins involved in phytohormone responses in *Arabidopsis*. **(E)** Number of genes encoding EAR motif-containing proteins involved in the stress response in *Arabidopsis thaliana*. **(F)** Number of genes encoding EAR motif-containing proteins associated with epigenetic modifications in *Arabidopsis thaliana*.

Using HMMER and inparanoid tools, we identified 17,567 EAR genes from 20,542 EAR motif-containing proteins across 71 plant species from algae to angiosperms (Figure 2A, Table 1). We compared the predicted results in *Oryza sativa, Sorghum bicolor, Vitis vinifera*, and *Populus trichocarpa* with those predicted in previous literature (Kagale et al., 2010) and found that our predicted results had a higher overlap with these species (Supplementary Figure 1). In addition, we also collected 25 reported EAR motif-containing proteins from the literature, and all of these reported EAR motif-containing proteins were included in our predictions (Supplementary Table 3). In addition, 771 GO and 132 KEGG terms were collected, which covered 13,839 and 1,479 EAR genes, respectively. We also collected 19,781 CDS, 16,360 transcript sequences and 20,542 protein sequences (Table 2, Supplementary Table 4). With the increasing availability of sequence data from non-model species, protein function in non-model plants can also be annotated by orthologous proteins in model plants based on sequence similarity. Here, we identified 21,920 pairs of orthologous EAR motif-containing proteins between *Arabidopsis thaliana* and other plants (Table 2, Supplementary Tables 4, 5).

**Table 1:** EAR motif-containing proteins distributions in all plants in plantEAR.

**Stage of evolution**	**Species**	**NO. of EAR proteins**	**NO. of all proteins with EAR patterns**	**EAR proteins/ proteins with EAR patterns**	**NO. of all proteins**	**EAR motif-containing protein/all protein**
Algae	*Dunaliella salina*	2	4262	0.05%	18801	0.01%
Algae	*Chlamydomonas reinhardtii*	4	7196	0.06%	19526	0.02%
Algae	*Micromonas pusilla CCMP1545*	11	1931	0.21%	10660	0.04%
Algae	*Volvox carteri*	4	5642	0.07%	16075	0.02%
Algae	*Micromonas* sp. *RCC299*	7	1899	0.37%	10103	0.07%
Algae	*Ostreococcus lucimarinus*	7	1321	0.53%	7796	0.09%
Algae	*Coccomyxa subellipsoidea C-169*	8	2338	0.34%	9629	0.08%
Bryophyta	*Sphagnum fallax*	107	9158	1.17%	32298	0.33%
Bryophyta	*Physcomitrella patens*	160	22420	0.71%	87533	0.18%
Bryophyta	*Marchantia polymorpha*	28	6370	0.44%	24674	0.11%
Pteridophyta	*Selaginella moellendorffii*	31	5799	0.53%	22285	0.14%
Gymnospermae	*Pinus taeda*	239	11707	2.04%	64809	0.37%
Gymnospermae	*Ginkgo biloba*	76	11347	0.66%	41840	0.18%
Gymnospermae	*Gnetum montanum Markgr*	99	6544	1.51%	27491	0.36%
Gymnospermae	*Pinus abies*	294	10817	2.72%	66632	0.44%
Basal Magnoliophyta	*Amborella trichopoda*	99	6152	1.61%	26846	0.37%
Angiosperms	*Glycyrrhiza uralensis*	328	10004	3.3%	38135	0.86%
Angiosperms	*Camellia sinensis*	204	8544	2.4%	36951	0.55%
Angiosperms	*Zostera marina*	115	5058	2.27%	20450	0.56%
Angiosperms	*Spirodela polyrhiza*	115	5023	2.29%	19623	0.59%
Angiosperms	*Panicum hallii*	195	13599	1.43%	49852	0.39%
Angiosperms	*Sorghum bicolor*	239	13477	1.77%	47205	0.51%
Angiosperms	*Setaria viridis*	240	13800	1.74%	48594	0.49%
Angiosperms	*Brachypodium distachyon*	247	15022	1.64%	52872	0.47%
Angiosperms	*Zea mays*	333	18460	1.80%	88760	0.38%
Angiosperms	*Musa acuminata*	393	9934	3.96%	36528	1.08%
Angiosperms	*Triticum aestivum*	394	59898	0.66%	293053	0.13%
Angiosperms	*Panicum virgatum*	474	28539	1.66%	125439	0.38%
Angiosperms	*Oryza sativa*	308	12595	5.92%	49061	1.52%
Angiosperms	*Amaranthus hypochondriacus*	134	5859	2.29%	23054	0.58%
Angiosperms	*Chenopodium quinoa*	148	12125	1.22%	44776	0.33%
Angiosperms	*Carica papaya*	151	5758	2.62%	27775	0.54%
Angiosperms	*Vitis vinifera*	163	6954	2.34%	26346	0.62%
Angiosperms	*Trifolium pratense*	174	9817	1.77%	41297	0.42%
Angiosperms	*Fragaria vesca*	175	9022	1.94%	32831	0.53%
Angiosperms	*Ricinus communis*	175	7218	2.42%	31221	0.56%
Angiosperms	*Daucus carota*	192	7885	2.44%	32118	0.60%
Angiosperms	*Prunus persica*	198	14161	1.40%	47089	0.42%
Angiosperms	*Mimulus guttatus*	202	8617	2.34%	33573	0.60%
Angiosperms	*Aquilegia coerulea*	203	11712	1.73%	41063	0.49%
Angiosperms	*Brachypodium stacei*	210	10318	2.04%	36357	0.58%
Angiosperms	*Citrus clementina*	219	9591	2.28%	33929	0.65%
Angiosperms	*Solanum lycopersicum*	226	7860	2.88%	34727	0.65%
Angiosperms	*Cucumis sativus*	229	8290	2.76%	30364	0.75%
Angiosperms	*Citrus sinensis*	231	12780	1.81%	46147	0.50%
Angiosperms	*Eucalyptus grandis*	238	13328	1.79%	46280	0.51%
Angiosperms	*Solanum tuberosum*	238	10192	2.34%	51472	0.46%
Angiosperms	*Phaseolus vulgaris*	285	9542	2.99%	31638	0.90%
Angiosperms	*Arabidopsis halleri*	301	7057	4.27%	26911	1.12%
Angiosperms	*Linum usitatissimum*	310	11747	2.64%	43484	0.71%
Angiosperms	*Eutrema salsugineum*	326	8046	4.05%	29284	1.11%
Angiosperms	*Arabidopsis lyrata*	348	8229	4.23%	32670	1.07%
Angiosperms	*Boechera stricta*	356	8194	4.34%	29812	1.19%
Angiosperms	*Malus domestica*	358	16829	2.13%	63517	0.56%
Angiosperms	*Capsella rubella*	362	8077	4.48%	28447	1.27%
Angiosperms	*Manihot esculenta*	371	11617	3.19%	41381	0.90%
Angiosperms	*Capsella grandiflora*	372	7263	5.12%	26561	1.40%
Angiosperms	*Medicago truncatula*	408	15156	2.69%	62319	0.65%
Angiosperms	*Brassica rapa FPsc*	428	10746	3.98%	41019	1.04%
Angiosperms	*Salix purpurea*	500	16889	2.96%	61520	0.81%
Angiosperms	*Populus trichocarpa*	518	19789	2.62%	73013	0.71%
Angiosperms	*Kalanchoe marnieriana*	521	19557	2.66%	69177	0.75%
Angiosperms	*Arabidopsis thaliana*	556	9681	5.74%	35386	1.57%
Angiosperms	*Glycine max Wm82*	564	21038	2.67%	73320	0.77%
Angiosperms	*Gossypium raimondii*	568	21418	2.65%	77267	0.74%
Angiosperms	*Gossypium arboreum*	500	10082	4.96%	40134	1.25%
Angiosperms	*Gossypium hirsutum*	982	18968	5.18%	70478	1.39%
Angiosperms	*Theobroma cacao*	241	13000	1.85%	44404	0.54%
Angiosperms	*Nicotiana tabacum Basma Xanthi*	791	21247	3.72%	86008	0.92%
Angiosperms	*Nicotiana tabacum K326*	728	21289	3.42%	85994	0.85%
Angiosperms	*Nicotiana tabacum TN90*	767	21924	3.50%	85439	0.90%
Angiosperms	*Nicotiana benthamiana*	522	14567	3.58%	76739	0.68%
Angiosperms	*Kalanchoe laxiflora*	292	12018	2.43%	45190	0.65%

**Table 2:** Data collection in PlantEAR.

**Content**	**Numbers**	**Source**
Species	71	–
EAR genes	17,567	Phytozome v12.0, PlantGenIE.org, SGN
EAR motif-containing proteins	20,542	Phytozome v12.0, PlantGenIE.org, SGN
CDS sequence	19,781	Phytozome v12.0, PlantGenIE.org, SGN
Transcript sequence	16,360	Phytozome v12.0, PlantGenIE.org, SGN
Protein sequence	20,542	Phytozome v12.0, PlantGenIE.org, SGN
Orthologous pairs	21,920	-
GO terms (genes)	771(13,839)	Phytozome v12.0, AmiGO 2
KEGG terms (genes)	132(1,479)	KEGG
Related publications	1650	PubMed, Tair
Gene with mutant phenotype	22	AHB2.0
Co-expression nodes/edges	51,516/247,274	ATTED-II, soyNET, RiceNet v2, TomotoNet, WheatNet, ccNET, SorghumFDB
Cis-element (motifs)	930	ccNET
Co-expression network modules	260	ccNET
Protein-Protein nodes/edges	35,160/147,233	BioGrid, TAIR, BAR, CCSB
Epigenetic modifications associated proteins	956	PCSD
Microarray Experiment-abiotic stress	248	GEO
Microarray Experiment-biotic stress	30	GEO
Microarray experiment-hormone	48	GEO
Microarray experiment-development	237	GEO

Following the iTAK (Zheng et al., 2016) rules, we carried out a gene family classification of EAR motif-containing proteins in each plant, and we identified 13,994 TFs and 3,073 TRs (Figure 2B, Supplementary Table 6). The 3,475 EAR motif-containing proteins were neither TFs nor TRs based on the iTAK software (Zheng et al., 2016; Figure 2B, Supplementary Table 6). In addition, EAR motif-containing proteins with unique EAR patterns were counted in all species, and we found that EAR motif-containing proteins with LxLxL, DNLx[1,2]P, and both patterns comprised 15,462, 2,212 and 2,868, respectively (Figure 2C, Supplementary Table 6).

Plant hormones can regulate numerous important biological processes in developmental and stress response processes. Several microarray expression profiles (Supplementary Table 2) were used to predict the stress or phytohormone response that EAR motif-containing proteins may involve in the model plant *Arabidopsis thaliana*. For analysis microarray with different time points for stress and hormonal responses, we called differentially expressed EAR motif-containing protein-coding genes at each time point, which may be involved in stress and hormones responses. As shown in Figures 2D, E, many EAR motif-containing proteins were significantly affected by phytohormones (Supplementary Table 7) and stress responses (Supplementary Table 8). In addition, 22 genes with morphological phenotypes controlled by plant hormones in *Arabidopsis thaliana* are also stored in plantEAR.

Epigenetic markers, such as DNA methylation, histone modifications and variants, can regulate gene expression by controlling chromatin structure. EAR motif-containing protein-coding genes that associated with epigenetic modifications, which were identified when epigenetic mark peaks located in the upstream of the transcription start site (TSS; 1 kb in *Arabidopsis*, 2 kb in rice and maize) or gene region of these genes were collected from 31 epigenomic data in the plant chromatin state database (PCSD; Liu et al., 2018). We obtained 401, 267, and 291 EAR genes associated with epigenetic modifications in *Arabidopsis thaliana, Oryza sativa* and *Zea mays*, respectively (Supplementary Table 9). For example, there were 292 EAR genes associated with H3K4me3 and 32 EAR genes associated with H3K9me2 in *Arabidopsis* (Figure 2F).

### Network analysis

The EAR motif-containing protein-coding genes perform their functions mainly by regulating downstream genes, so it was necessary to integrate or construct networks (co-expression or PPI network) to analyse downstream genes that might be regulated by the EAR motif-containing proteins.

#### Co-expression network

Gene co-expression networks are usually constructed using transcriptome data. Through integrating the co-expression network of 9 plant species, we produced an EAR gene co-expression network of 51,516 nodes and 247,274 edges (Table 2, Supplementary Table 4). Co-expression network analysis is powerful for predicting gene functions. Genes in the same co-expression network have similar expression patterns and may be involved in the same biological processes. The function of an EAR motif-containing protein can be predicted by its well annotated co-expressed genes, so the integration of co-expression networks will benefit the study of the function of EAR motif-containing coding genes. In addition, co-expression network with an expression view and expression heatmap of *Arabidopsis thaliana* is also offered, as well as *Gossypium hirsutum* and *Gossypium arboreum*.

Co-expression network comparison analysis between species is a way to illustrate the conservation and differences of orthologous gene co-expression networks. *Arabidopsis* is currently the most studied model plant, and its functional annotations are more comprehensive. Cotton is an important dicotyledonous plant, and its functional annotation is still quite lacking. If the co-expression network of *Arabidopsis* EAR genes and cotton orthologous EAR genes is highly conserved, we can use the well annotated *Arabidopsis* genes or co-expressed genes to predict the function of poorly annotated cotton genes. Here, co-expression networks of orthologous EAR gene pairs between *Arabidopsis thaliana* and two types of cotton, including *Gossypium hirsutum* and *Gossypium arboreum*, was used to make comparisons, and the results are easily accessible from plantEAR.

Functional modules are genes that are closely related in a co-expression network. The functional similarity of these genes is higher than that of co-expression networks. In a module, the function of unknown genes can also be reflected by genes with known function. Functional modules may also contribute to the mining of function of EAR motif-containing protein-coding genes. By using Cfinder software, 27 co-expression functional modules in *Arabidopsis* were predicted. In addition, 233 functional modules of EAR motif-containing protein-coding genes and corresponding GSEA annotations for *Gossypium hirsutum* and *Gossypium arboreum* were also collected from ccNET (Table 2, Supplementary Table 4; You et al., 2017).

#### Predicted PPI network

Protein-protein interactions (PPIs) are an essential part of plant signal transduction or transcriptional regulation. The identification of potential PPIs in non-model plants may provide better research capabilities for the identification of conserved functions of EAR motif-containing proteins. Basing on the putative orthologous relationship predicted by inparanoid software between *Arabidopsis thaliana* and other species, we obtained an EAR motif-containing protein special PPI network of 35,160 nodes and 147,233 edges (Table 2, Supplementary Table 4). In addition, PPI networks with expression view and expression heatmap of *Arabidopsis thaliana, Gossypium hirsutum*, and *Gossypium arboreum* are also offered in plantEAR.

### Functional enrichment analysis tools

In addition to the co-expression and PPI network that can be used to predict the function of EAR motif-containing proteins, two functional enrichment analysis tools have been developed for gene annotation, including gene set enrichment analysis (GESA) and cis-element enrichment analysis. GSEA for 15 plant species and cis-element enrichment analysis for 4 species are offered in this platform.

### Implementation

**Figure 3:**
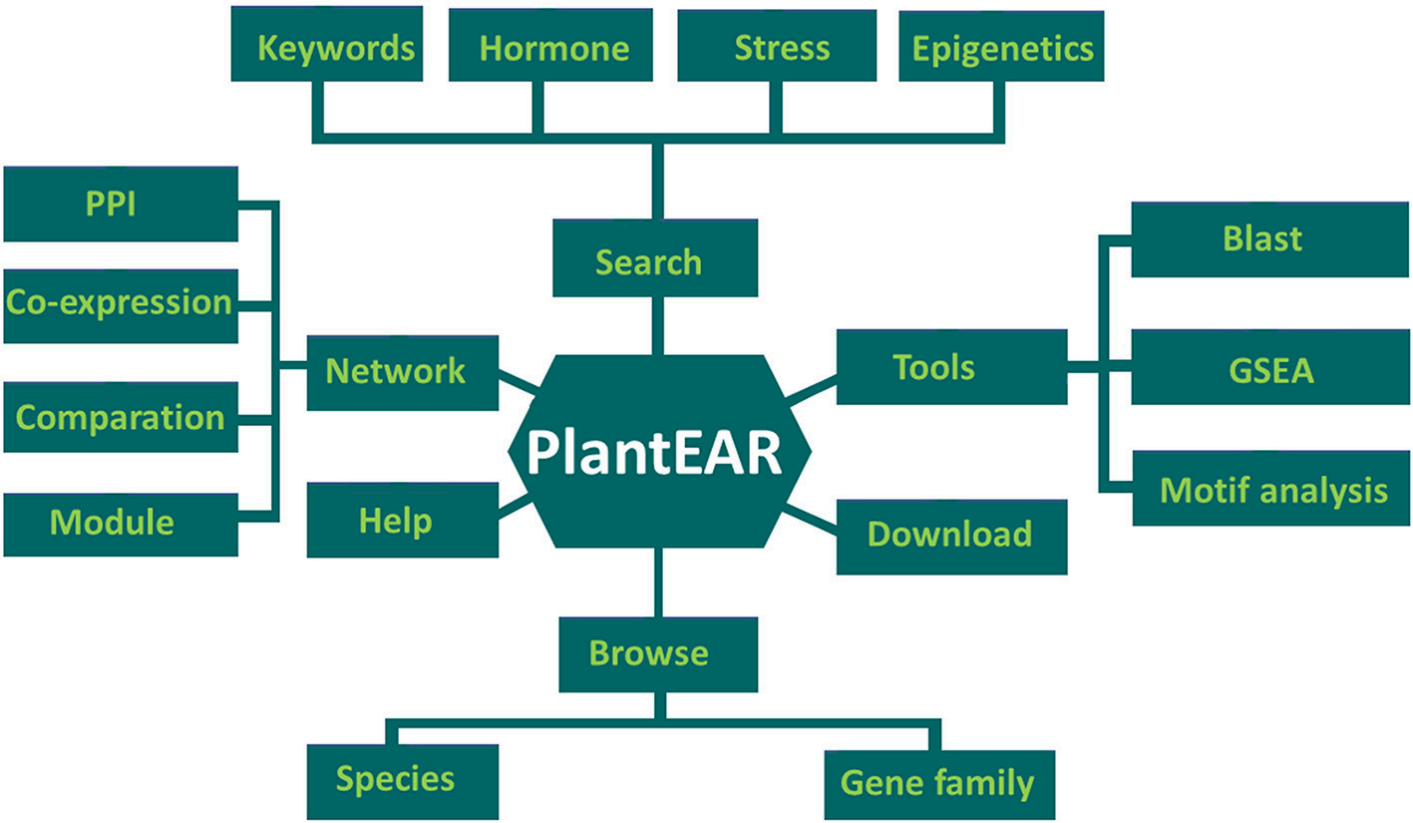
The structure of plantEAR, including six sections: search section, browse section, tools section, download section, network section, and help section.

Based on our data collection and functional annotations, the EAR motif-containing protein functional analysis platform (PlantEAR) was constructed under the classical LAMP (Linux + Apache + Mysql + PHP) environment. There are six sections in plantEAR, including Search, Tools, Download, Browse, Network, and Help (Figure 3). The Search tool contains the keyword search, search for EAR motif–containing proteins related to different hormones, stress, and epigenetic modifications. The tools include BLAST search, gene set enrichment analysis (GSEA), and cis-element (motif) analysis. The download page can allow the download of EAR motif-containing protein-related sequence information, gene family, EAR pattern, and annotation information for various species. The EAR motif-containing proteins can be browsed by species and family classification, and we also provide help information for users using the functional analysis platform. The network section contains co-expression networks, protein-protein interaction networks and functional modules for EAR motif-containing proteins (Figure 3).

## Application

### The functional analysis of the EAR motif-containing protein *RGL3*

**Figure 4:**
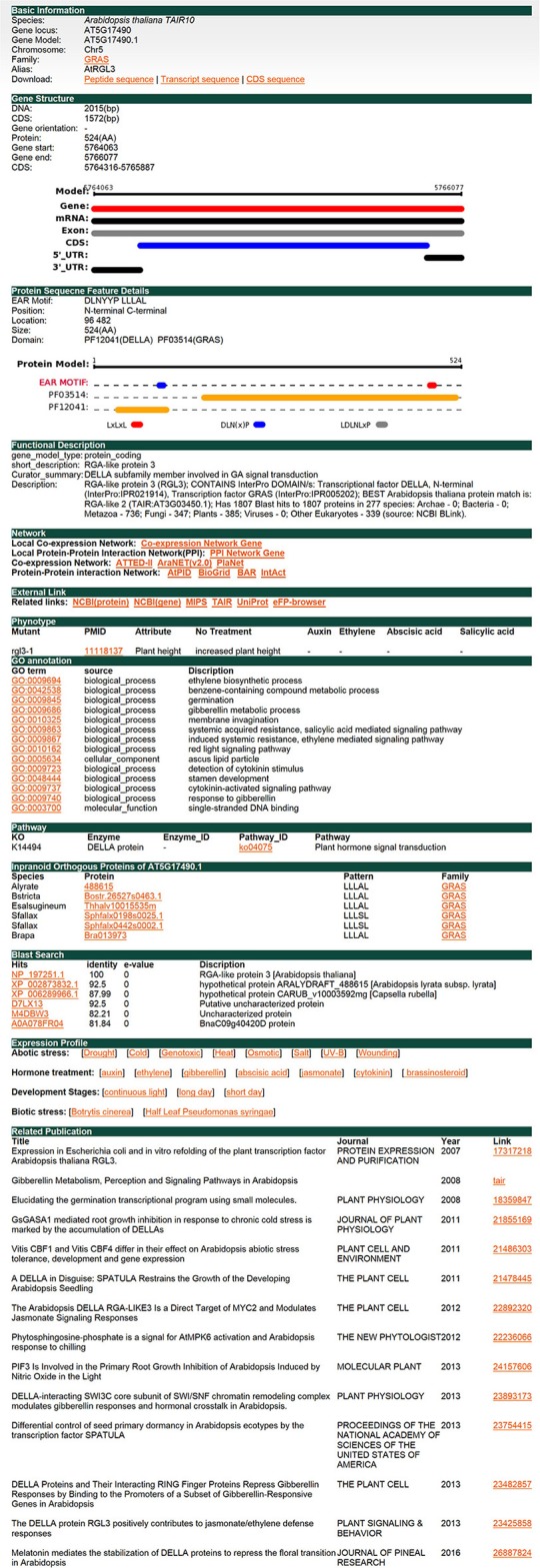
Individual gene detail page. Here, we use a protein RGL3 from the GRAS family as an example. This figure shows separate web pages where users can find detailed information, such as gene structure, protein sequence features, functional description, internal and external related links, reported phenotype, GO annotation, KO annotation, orthologs, expression profiles, and related publications.

**Figure 5:**
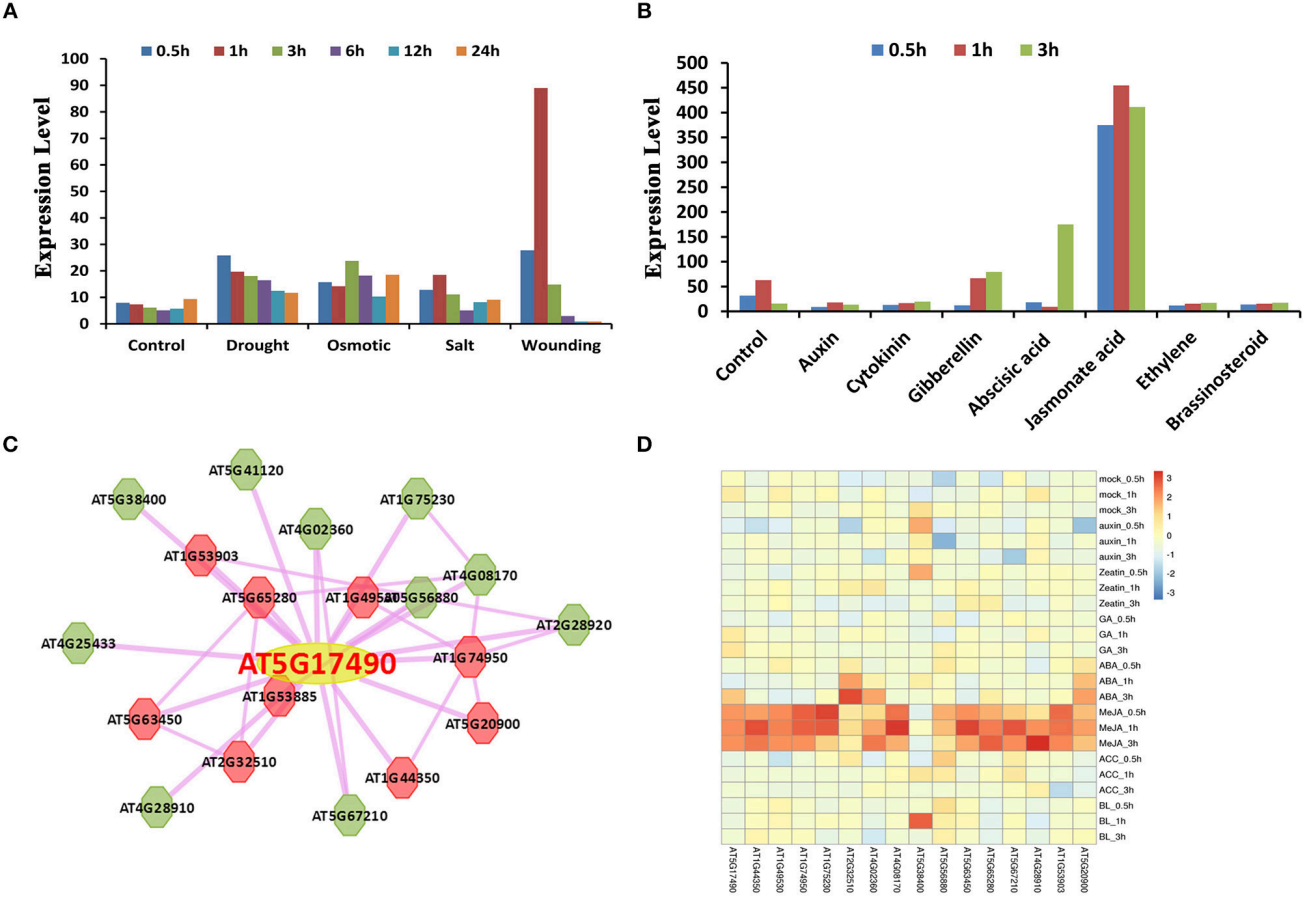
Example of the application of plantEAR. **(A)** The expression patterns of *RGL3* under drought, osmotic, salt and wounding stress in *Arabidopsis* root. **(B)** The expression patterns of *RGL3* treated with jasmonic acid, abscisic acid, gibberellin, etc. **(C)** Co-expression network expression view analysis of *RGL3* under 3 h of salt stress. **(D)** Co-expression heatmap analysis of *RGL3* under hormone treatment.

*RGA-LIKE PROTEIN 3* (*RGL3*) is a protein of the GRAS family. It has been reported that RGL3 is involved in the response of abscisic acid (ABA) and gibberellins (GA). RGL3 was upregulated under ABA treatment and downregulated under PCA, an inhibitor of GA synthesis, treatment (Piskurewicz and Lopez-Molina, 2009). RGL3 is the target gene of MYC2 and participates in the jasmonate (JA) response in a CORONATINE INSENSITIVE1 (COI1) and JASMONATE INSENSITIVE1 (MYC2)-dependent manner (Wild et al., 2012). In addition, RGL3 is also involved in salt stress resistance (Shi et al., 2017). Here we used *RGL3* in *Arabidopsis thaliana* as an example to illustrate the function offered in plantEAR (Figure 4). The annotation information in the gene detail page indicates that the protein belongs to the GRAS family, which has two EAR motifs, DLNYYP and LLLAL, located in the DELLA and GRAS domains, respectively. The functional description suggests that *RGL3* is involved in the signaling pathway of GA. The local co-expression network link, PPI network link, external link, and phenotype are also available. GO and KEGG annotation are also available, which suggest that *RGL3* is involved in phytohormone response processes, such as GA, SA, and ethylene. Orthologous proteins of *RGL3* in many species also have similar motif patterns, suggesting that EAR motifs of RGL3 may be conserved in different species (Figure 4). Expression profiles, BLAST search results, and relevant literature information are also provided in plantEAR. In addition, expression profiles are easily accessible in plantEAR. Based on the expression profiles, we found that *RGL3* was significantly upregulated under abiotic stress in *Arabidopsis thaliana* roots (Figure 5A), such as drought stress, salt stress, osmotic, and wounding conditions. Similarly, the gene is also upregulated in response to the relevant hormone treatment, such as jasmonic acid, abscisic acid, and gibberellin (Figure 5B).

There are 19 co-expressed genes (Supplementary Table 10) and 26 interacting proteins (Supplementary Table 11) of *RGL3*. Several *RGL3* co-expressed genes were upregulated under 3 h of salt stress and hormone treatment (Figures 5C,D), as well as genes in its PPI network (Supplementary Figures 2A,B). Heatmap analysis of genes in the *RGL3* co-expression and PPI network suggested that *RGL3* is involved in hormone and stress responses (Supplementary Figures 2B–D). Co-expressed genes of *RGL3* were then selected for GSEA analysis using default parameters (Fisher's exact test, *P* ≤ 0.05), and gene sets related to jasmonic acid and auxin were enriched (Supplementary Table 12). Cis-element significant enrichment analysis was performed to analyse the co-expressed genes of *RGL3*, and the results suggested that motifs related to gibberellin, ABA and jasmonate were also significantly enriched (Supplementary Table 13). In addition, GSEA analysis of 26 interacting proteins of *RGL3* showed significantly enriched gene sets related to hormones (abscisic acid, gibberellin, and jasmonic acid) and the stress response (dehydration stress, drought stress, salt stress and oxidative stress; Supplementary Table 14). These results indicate that *RGL3* may be involved in the response of abscisic acid, jasmonic acid, gibberellin and salt, drought, oxidative and osmotic stress, etc.

We searched the functional modules of *RGL3* through plantEAR. We found that *RGL3* is contained in a module of four genes (RGL3, CYP94B1, MAPKKK17, and GCL1), while CYP94B1 is involved in the response of jasmonic acid and wounding (Poudel et al., 2016). Hormone-related heatmap analysis also indicated that the module might be involved in the response process of jasmonic acid and ABA (Supplementary Figure 2E). Stress-related heatmap analysis indicated that the module might be involved in processes such as wounding and drought stress (Supplementary Figure 2F). Therefore, these results indicate that *RGL3* may have multiple functions.

Through functional annotations and functional enrichment analysis, we found that *RGL3* responded to several hormones and stresses in *Arabidopsis* (Figure 5). *RGL3* responses to gibberellin, ABA, JA and salt stress have been verified (Piskurewicz and Lopez-Molina, 2009; Wild et al., 2012; Shi et al., 2017), but several inferred functions are still unknown, such as drought, oxidative and osmotic stress responses. These predicted but unknown functions of RGL3 can provide reference or direction for related research in the future.

### The functional analysis of EAR motif-containing protein *ERFs* in *arabidopsis*

In the ERF family, *ERF3, ERF4*, and *ERF7* are EAR motif-containing proteins. *ERF3* has been reported to be involved in plant salt stress responses (Song and Galbraith, 2006). *ERF4* is involved in the response process of ethylene and ABA (Yang et al., 2005), and *ERF7* is involved in the response of ABA and drought stress (Song et al., 2005). We found that ERF3 was upregulated under salt stress in roots (Supplementary Figure 3A), and the three genes in the co-expression network were also upregulated under 6 h of salt stress (Supplementary Figure 3B). The expression of *ERF4* was upregulated by ABA treatment (Supplementary Figure 3C), and two genes in its co-expression network were also upregulated by ABA treatment at 0.5 h (Supplementary Figure 3D). In addition, GSEA analysis of co-expressed genes of ERF4 showed significantly enriched gene sets related to ethylene response, cell communication, the defense response, and carbohydrate stimulus (Supplementary Table 15). Under conditions of ABA treatment for 3 h (Supplementary Figure 3E) and drought stress (Supplementary Figure 3F), the expression of *ERF7* was also upregulated. The functions of these ERFs analyzed by the method provided by plantEAR are consistent with previously reported functions (Song et al., 2005; Yang et al., 2005; Song and Galbraith, 2006). These results show that the PlantEAR is beneficial for exploring the functions of plant EAR motif-containing proteins.

### The network comparison analysis of the EAR motif-containing protein *JAZ5*

**Figure 6:**
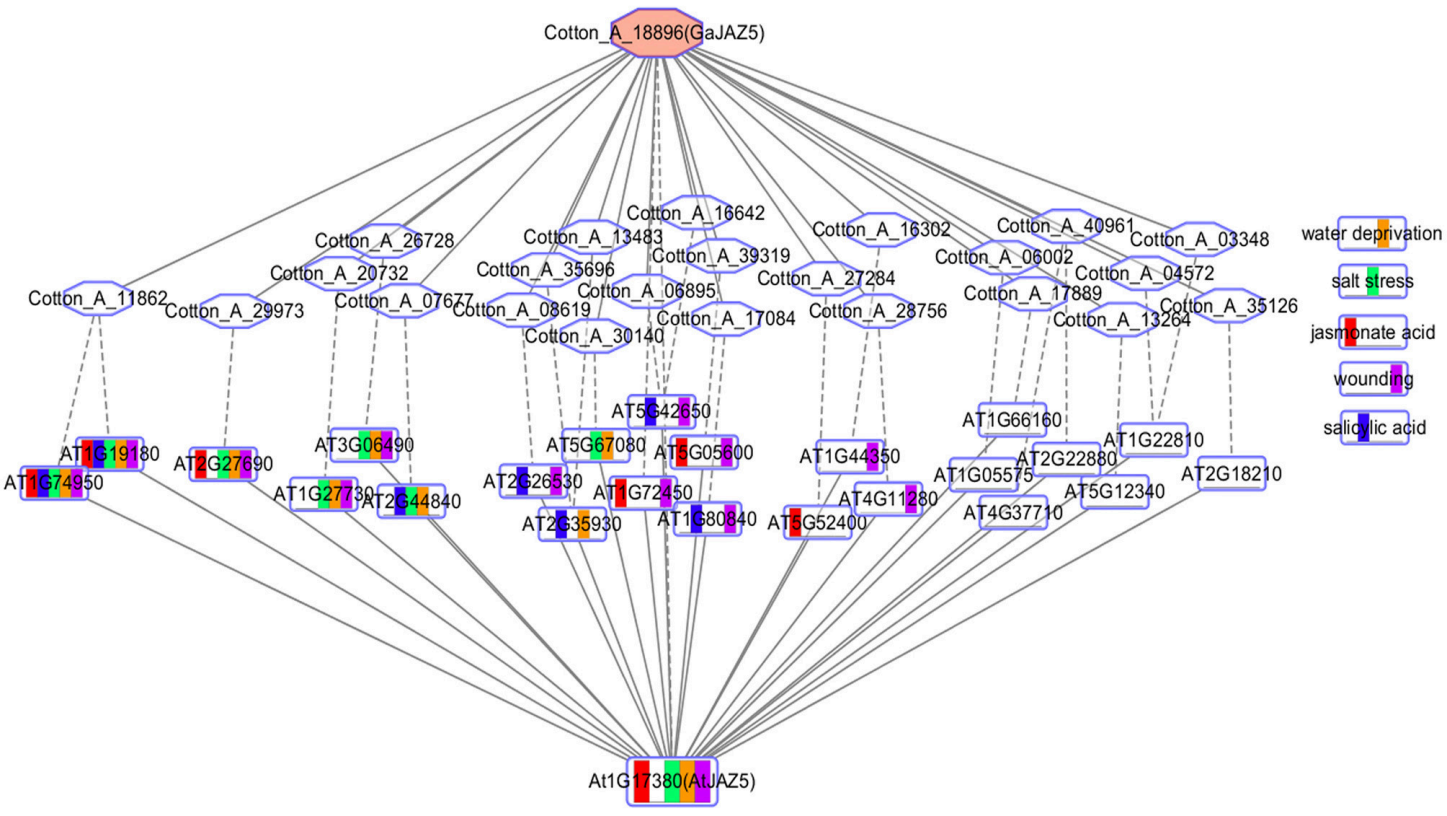
Network comparison between *AtJAZ5* and *GaJAZ5*. The rectangle and hexagon represent the genes of *Arabidopsis thaliana* and *Gossypium arboreum*, respectively. Red represents the genes that respond to jasmonic acid, green represents genes that respond to salt stress, blue represents genes that respond to salicylic acid, yellow represents genes that respond to water deprivation, and purple represents genes that respond to wounding. The rectangle with multiple colors represents genes annotated by multiple functions. The solid line connects two co-expressed genes, and the dotted line connects two orthologous genes.

The conserved co-expression network between different species may reveal conserved functions of orthologous genes. Co-expression networks typically use Pearson correlation coefficient (PCC) or mutual rank (MR) to the define thresholds. We obtained the co-expressed genes of the PCC top 300 of *Gossypium arboreum* and *Gossypium hirsutum* EAR genes from ccNET and the top 300 MR co-expressed genes of *Arabidopsis* EAR genes from ATTED-II. Here, we take the EAR gene *GaJAZ5* (Cotton_A_18896) of *Gossypium arboreum* as an example and compare its top 300 PCC co-expression network with the top 300 MR co-expression network of its orthologous gene *AtJAZ5* in *Arabidopsis thaliana* (Figure 6). There are 27 orthologous gene pairs, including 24 genes in *Arabidopsis* and 24 genes in *Gossypium arboreum*, as shown in Figure 6. Twenty-four genes in *Arabidopsis* were selected for GSEA analysis, and gene sets related water deprivation, salt, wounding, jasmonic acid, and salicylic acid responses were significantly enriched. Drought and salt can lead to water deprivation, while *GaJAZ5* overexpression in *Arabidopsis thaliana* can improve drought tolerance (Zhao et al., 2016). However, *GaJAZ5* functions in salt stress, jasmonate acid, and salicylic acid responses remain to be explored. The function of *GaJAZ5* can be predicted by the *Arabidopsis AtJAZ5* co-expression network, suggesting that the comparison of co-expression networks between species is beneficial for gene function prediction in non-model plants.

### Phylogenetic analysis

**Figure 7:**
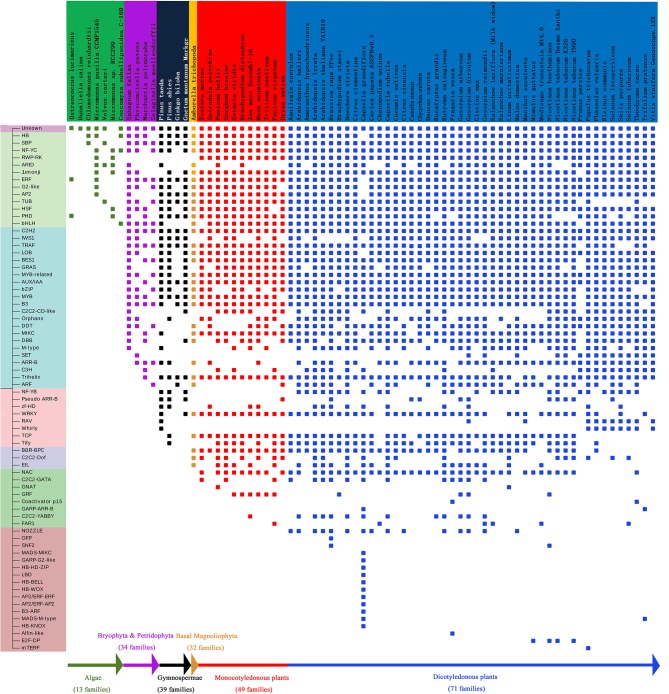
Analysis of gene families of EAR motif-containing proteins. The vertical axis represents the gene family. Different colors on the horizontal axis represent species at different stages of evolution. Small rectangles represent the existence of gene families of EAR motif-containing proteins in different species.

In plant evolutionary processes, statistical analysis suggests that the average number of EAR motif-containing proteins shows a tendency to increase in different evolutionary stages, as well as the proportion of EAR motif-containing proteins (Figure 2A). The number of gene families containing EAR genes is also increased in different evolutionary stages (Figure 7). There are 13 gene families in algae, 34 gene families in Pteridophyta and Bryophyta, 39 gene families in gymnosperms, 49 gene families in monocotyledonous plants and 71 gene families in dicotyledonous plants (Figure 7). For gene families with EAR motif-containing proteins, once the EAR motif-containing proteins begins to appear in a certain family, then the later advanced evolutionary stages will not disappear. For example, from bryophytes to angiosperms, the AUX/IAA family always contains EAR motif-containing protein members. From algae to angiosperms, the homeobox (HB) family always contains EAR motif-containing proteins (Figure 7). The Tify family always has EAR motif-containing proteins from gymnosperms (Figure 7).

**Figure 8:**
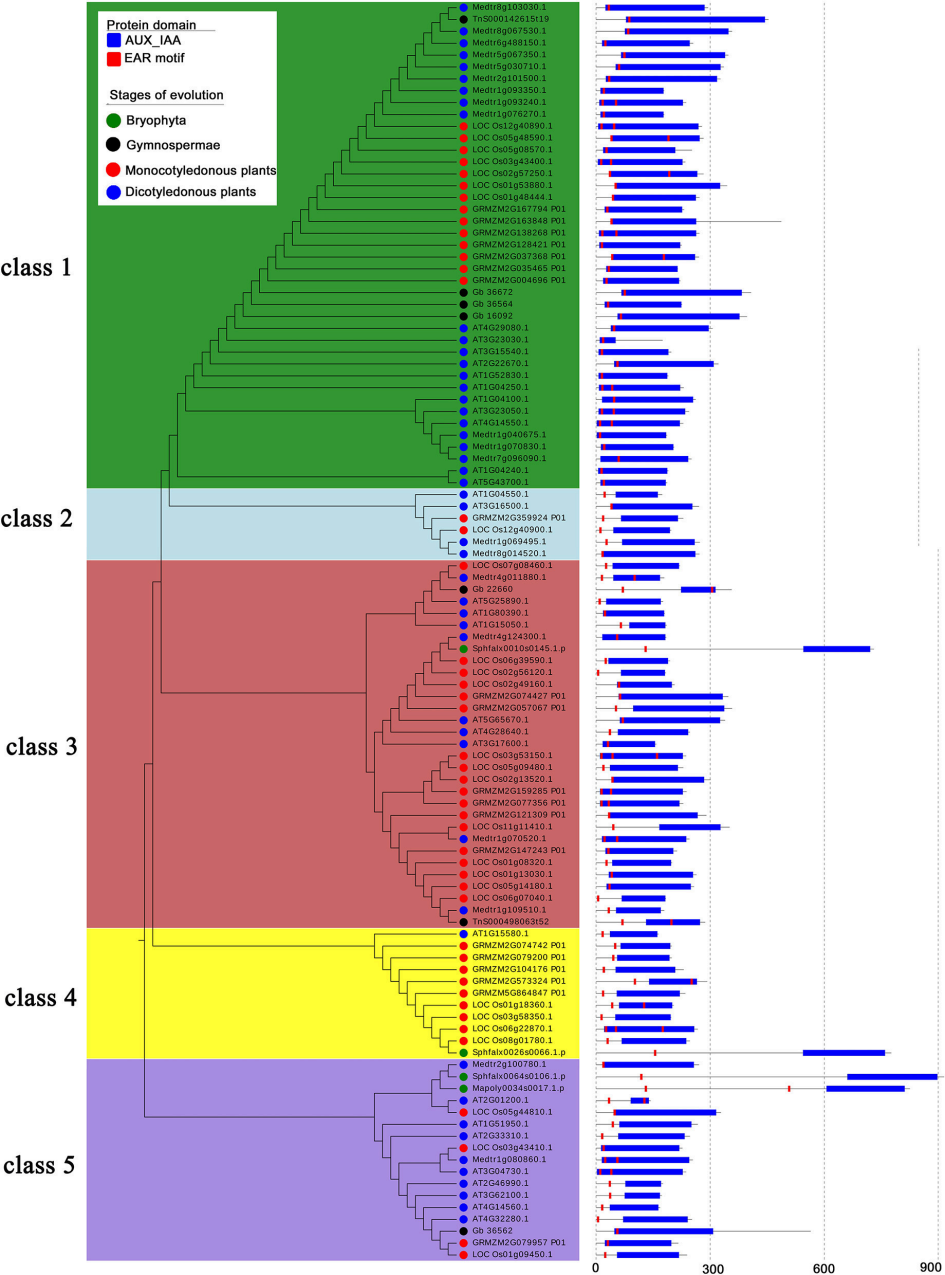
Phylogenetic tree of EAR motif-containing proteins in the AUX/IAA family across different species. Protein sequences of eight species from four evolutionary stages were chosen to construct the unrooted tree, which was divided into five classes. The blue rectangle represents the conserved domain of AUX/IAA, and the red rectangle represents the location of the EAR motif.

We found that the EAR motif-containing proteins in the AUX/IAA family originally appeared in bryophytes (Figure 8). Therefore, we chose conserved regions of the protein sequences in two bryophytes (*Sphagnum fallax* and *Marchantia polymorpha*), two gymnosperms (*Ginkgo biloba* and *Gnetum montanum Markgr*), two monocotyledons (*Zea mays* and *Oryza sativa*), and two dicotyledonous (*Arabidopsis thaliana* and *Medicago truncatula*) to construct the phylogenetic tree. The EAR motif-containing proteins of the AUX/IAA family can be classified into five classes, among which the EAR motif-containing proteins of bryophytes are contained in the third, fourth, and fifth class, while no bryophyte EAR motif-containing proteins are contained in the first and second class (Figure 8). For branches with bryophyte EAR motif-containing proteins, most of the EAR motifs are located in the N-terminus of the proteins. For branches without bryophyte EAR motif-containing proteins, the EAR motif is contained in the IAA domain in the first branch. These results may indicate that the AUX/IAA family EAR motif-containing proteins have been amplified after the bryophytes stage (Figure 8).

We also found that the EAR motif-containing proteins of the HB family originated from algae (Supplementary Figure 4). Two algae (*Micromonas pusilla CCMP1545, Chlamydomonas reinhardti*i), two ferns (*Sphagnum fallax, Physcomitrella patens*), two gymnosperms (*Ginkgo biloba, Gnetum montanum Markgr*), one basal angiosperms (*Amborella trichopoda*), two monocots (*Oryza sativa* and *Sorghum bicolor*), and two dicots (*Arabidopsis thaliana* and *Medicago truncatula*) were used to construct a phylogenetic tree (Supplementary Figure 4). The HB phylogenetic tree can be divided into five classes. Class 1 and Class 5 are branches containing algae. Therefore, the other branches may be newly generated branches of EAR motif-containing proteins in plant evolution. The outermost protein structure features indicate that the structures of the EAR motif-containing proteins in the same class are similar (Supplementary Figure 4). Therefore, the EAR motif-containing proteins in the HB family may be conservative in evolution.

We also found that Tify EAR motif-containing proteins may have initially appeared in gymnosperms. Thus, one gymnosperm (*Pinus abies*), two monocotyledons (*Sorghum bicolor* and *Oryza sativa*), and two dicotyledonous (*Arabidopsis thaliana* and *Gossypium hirsutum*) were selected to construct the phylogenetic tree (Supplementary Figure 5). We found that EAR motif-containing proteins in Tify family were conserved in branch one and seven, so the EAR motif-containing proteins of the Tify family are conserved to some extent.

## Discussion

Transcriptional repression is a part of transcriptional regulation that plays an important role in plant growth and development as well as environmental adaptation. EAR motif-mediated transcriptional repression is the main representative of transcriptional repression (Kagale and Rozwadowski, 2011; Causier et al., 2012; Ma et al., 2017; Wu and Citovsky, 2017; Choi et al., 2018), so prediction and functional annotation for EAR motif-containing proteins are required for studying transcriptional repression. Here, HMMER in combination with inparanoid was used to predict plant EAR motif-containing proteins. For Arabidopsis, we have tested the sensitivity of this workflow preliminary. We randomly selected 70% of 219 high-confidence EAR motif-containing proteins as the initial seeds for HMM construction, with E value as 0.01 for 10 repetitions, the average predicted result covers 91% of the total 219 proteins, as well as 30 additional proteins (outside the 398 Arabidopsis EAR motif-containing proteins, probably false positive predictions). In addition, the predictions of the four species also covered most of the previous predictions (Supplementary Figure 1), as well as several reported EAR motif-containing proteins (Supplementary Table 3). Therefore, the method we use are acceptable and usable for predicting plant EAR motif-containing proteins. We hope we could improve the assessment of our method with more validated EAR motif-containing proteins collected.

A previous study suggests that EAR motif-containing proteins in *Arabidopsis* can be grouped into transcription factor (TF) and other transcriptional regulator (TR) families (Kagale et al., 2010). Here, 20,542 EAR motif-containing proteins of 71 plants were identified, 83% of which are members of TF/TR families. This finding indicated that the EAR motif-containing proteins perform their functions mainly by transcriptional regulation as described (Kagale et al., 2010). A total of 17% of predicted EAR motif-containing proteins may not be transcription factors or transcriptional regulators, but they are still involved in the transcriptional regulation process (Figure 2B), such as *KIX8*/*KIX9*, which are involved leaf growth regulation in *Arabidopsis thaliana* (Gonzalez et al., 2015). This phenomenon suggests that there may be many undescribed transcriptional repression factors involved in the transcriptional regulation process. In addition, transcriptome analysis in *Arabidopsis thaliana* was used to evaluate the special reactions of EAR motif-containing proteins to external signals. These detailed annotations of the family classification and specific functions of EAR motif-containing proteins may provide directions for researchers to study EAR motif-mediated transcriptional repression.

PlantEAR may promote the functional study of plant EAR motif-containing proteins. The network expression view as part of the network analysis can optimize deficiencies of the common network analysis. An example is shown by the results of the functional analysis of *RGL3*, many functions of which have been experimentally verified (Piskurewicz and Lopez-Molina, 2009; Wild et al., 2012; Shi et al., 2017). Co-expression network comparative analysis is also a part of network analysis. We performed a comparative analysis of the *GaJAZ5* and *AtJAZ5* co-expression network, which can well demonstrate the advantages of network comparison and reflect the conservation of co-expression networks of *GaJAZ5* and *AtJAZ5*. Functional enrichment analysis is very important for studying the function of a gene list in the network. Therefore, functional annotations, network analysis, and functional enrichment analysis are special features of PlantEAR.

Phylogenetic analysis across species shows that EAR motif-containing proteins may have a positive effect on the evolutionary selection of plants. In the process of evolution, plants will not be naturally eliminated if they constantly change their ability to adapt to the environment. There are many adverse factors in the natural environment that can affect the survival of plants, such as drought stress and salt stress. Many reported plant EAR motif-containing proteins are involved in processes such as stress response, growth and hormonal responses, which may have effects on plant adaptation to environmental changes. Therefore, to adapt to the environment during the natural selection process, plants may mutate or produce some new EAR motif-containing protein-encoding genes, thereby increasing their ability to resist stress and allowing the plants to survive the natural selection process. EAR motif-containing proteins appeared in algae and showed remarkable expansion in the evolutionary stage of moss/ferns (Figure 2A), which may play roles in plant evolution from aquatic to terrestrial plants. From moss/ferns to gymnosperms and then to angiosperms, EAR motif-containing proteins also significantly increased, as well as gene families with EAR motif-containing proteins (Figure 7), which may indicate that EAR motif-containing proteins contribute to plant evolution. The evolutionary analysis of EAR motif-containing proteins in the IAA/AUX, HB, Tify family may indicate that EAR motif-containing proteins may be somewhat conserved, which is consistent with a previous report (Kagale et al., 2010).

PlantEAR provides a variety of clues to study the functions or evolution of proteins with EAR motifs, but the EAR motif-containing proteins of many species are still unpredicted. Therefore, predictions and functional annotations of EAR motif-containing proteins for more species still need to be performed, which will benefit studies on the function and evolution of EAR motif-containing proteins in more species.

## Conclusions

PlantEAR is the first analysis platform for plant EAR motif-containing proteins, and it contains 20,542 predicted EAR motif-containing proteins across 71 plants. PlantEAR provides resources for the study of EAR motif-mediated transcriptional repression, including functional annotations, sequence information, gene families, and related publications. In addition, several analytic tools in plantEAR can be used to perform more in-depth functional mining of EAR motif-containing proteins, such as network analysis and gene set enrichment analysis. In the future, we will continue to adjust our methods to predict EAR motif-containing proteins based on new reports. We will also perform long-term maintenance and species updating. We hope PlantEAR will facilitate the study of plant transcription repression.

## Author contributions

This study was designed by ZS and WX. The platform construction and draft were completed by JY. The epigenetic modifications data was processed by YL. QY, LZ, and HY provided technical supports. ZS, WX, and TT participated in the modification of draft.

### Conflict of interest statement

The authors declare that the research was conducted in the absence of any commercial or financial relationships that could be construed as a potential conflict of interest.
