# Supplementary material for: PlantEAR: Functional Analysis Platform for Plant EAR Motif-Containing Proteins
Source: Front Genet. 2018 Nov 30;9:590. doi: 10.3389/fgene.2018.00590 (PMC6283911; doi:10.3389/fgene.2018.00590)
Supplement: Supplementary file 1 [file Table_1.doc]

Supplementary Material

**PlantEAR: Functional Analysis Platform for Plant EAR Motif-Containing Proteins**

**Jiaotong Yang, Yue Liu, Hengyu Yan, Tian Tian, Qi You, Liwei Zhang, Wenying Xu*and Zhen Su***

***To whom correspondence should be addressed.**

**Zhen Su (**[zhensu@cau.edu.cn](mailto:zhensu@cau.edu.cn)**); Wenying Xu (**[xwenying@hotmail.com](mailto:xwenying@hotmail.com)**)**

# Supplementary Figures and Tables

## Supplementary Tables

| **Table S1.** Resource for plantEAR. | | | |
| --- | --- | --- | --- |
| **Class** | **Platforms** | **Resource** | **Link** |
| Genome data | Phytozome v12.0 | Plant Comparative Genomics Resource of mutiplt plants | <https://phytozome.jgi.doe.gov/pz/portal.html> |
|  | PlantGenIE.org | The Plant Genome Integrative Explorer Resource | <http://plantgenie.org/> |
|  | GIGAdb | De novo sequencing of Ginkgo biloba | http://gigadb.org/dataset/100209 |
|  | SGN | The Sol Genomics Network--from genotype to phenotype to breeding | <https://solgenomics.net/> |
|  | DRYAD | Open data sources for scientific publications discoverable | https://datadryad.org/resource/doi:10.5061/dryad.0vm37 |
| Annotation data | Tair | The Arabidopsis Information Resource | <http://www.arabidopsis.org/> |
|  | PlantTFDB v4.0 | Plant Transcription Factor Database | <http://planttfdb.cbi.pku.edu.cn/> |
|  | iTAK | Plant Transcription factor & Protein Kinase Identifier and Classifier | <http://bioinfo.bti.cornell.edu/cgi-bin/itak/index.cgi> |
|  | Pfam | A large collection of protein families, each represented by multiple sequence alignments and hidden Markov models (HMMs) | <http://pfam.xfam.org/> |
|  | AHD v2.0 | Arabidopsis hormone database v2.0 | <http://ahd.cbi.pku.edu.cn/> |
|  | AmiGO 2 | Stardard Gene Ontologies Description | <http://amigo.geneontology.org/amigo/landing> |
|  | KEGG | Kyoto Encyclopedia of Genes and Genomes | <http://www.kegg.jp/> |
| Coexpression network | SorghumFGD | Sorghum functional genomics database | <http://structuralbiology.cau.edu.cn/sorghum/> |
|  | ATTED-II | A database of co-expressed genes and cis elements for identifying co-regulated gene groups in Arabidopsis. | <http://atted.jp/> |
|  | RiceNet v2 | An improved network prioritization server for rice genes | <http://www.inetbio.org/ricenet/> |
|  | WheatNet | Gene network of bread wheat | <http://www.inetbio.org/wheatnet/> |
|  | SoyNet | Gene network and coexpression network database for soybean | <http://www.inetbio.org/soynet/> |
|  | TomatoNet | Co-functional network of Tomato | <http://www.inetbio.org/tomatonet/> |
|  | ccNET | co-expression networks with functional modules for diploid and polyploid Gossypium | <http://structuralbiology.cau.edu.cn/gossypium/> |
|  | PlaNET | A platform of web-tools dedicated to visualization and analysis of plant co-function networks | <http://aranet.mpimp-golm.mpg.de/> |
|  | MCENet | A database for maize conditional co-expression network and network characterization collaborated with multi-dimensional omics levels | http://bioinformatics.cau.edu.cn/MCENet/ |
|  |  |  |  |
| PPI network | AtPID | Arabidopsis thaliana Protein Interactome Database | <http://www.megabionet.org/atpid/webfile/> |
|  | BioGrid | An interaction repository with data compiled through comprehensive curation efforts. | <https://thebiogrid.org/> |
|  | CCSB | Plant interaction database | <http://interactome.dfci.harvard.edu/A_thaliana/> |
|  | BAR | The Bio-Analytic Resource for Plant Biology | <http://bar.utoronto.ca/> |
| Transcriptome data | GEO | Gene Expression Omnibus | <https://www.ncbi.nlm.nih.gov/geo/> |
| Epigenomic data | PCSD | Plant Chromotin state database | <http://systemsbiology.cau.edu.cn/chromstates/> |

**Table S2.** List of microarray samples used in plantEAR

**Table S3.** Reported EAR motif-containing proteins in plants (besides *Arabidopsis*)

**Table S4.** Data collection for plantEAR

**Table S5.** Ortholgous relationship of EAR motif-containing proteins

**Table S6.** List of predicted EAR motif-containing proteins in different plant species

**Table S7.** Phytohormone response genes that encoding EAR motif-containing proteins in *Arabidopsis*

**Table S8.** Stress response genes that encoding EAR motif-containing proteins in *Arabidopsis*

**Table S9.** List of EAR motif-containing protein-coding genes that associated with epigenetic modifications in *Arabidopsis thaliana*, *Zea mays* and *Oryza sativa*

(Table S2-S9 in separated excel file)

| **Table S10.** Co-expressed genes of RGL3 in *Arabidopsis.* | | |
| --- | --- | --- |
| **Gene** | **Alias** | **Annotation** |
| AT2G28920 |  | RING/U-box superfamily protein |
| AT4G25433 |  | Peptidoglycan-binding LysM domain-containing protein |
| AT5G20900 | JAZ12,TIFY3B | Jasmonate-zim-domain protein 12 |
| AT1G53885 |  | Protein of unknown function (DUF581) |
| AT1G53903 |  | Protein of unknown function (DUF581) |
| AT5G41120 |  | Esterase/lipase/thioesterase family protein |
| AT4G28910 | NINJA | Novel interactor of JAZ |
| AT5G67210 | IRX15-L | Protein of unknown function (DUF579) |
| AT5G65280 | GCL1 | GCR2-like 1 |
| AT5G63450 | CYP94B1 | Cytochrome P450, family 94, subfamily B, polypeptide 1 |
| AT5G56880 |  | Hypothetical protein |
| AT5G38400 |  | Hypothetical protein |
| AT4G08170 |  | Inositol 1,3,4-trisphosphate 5/6-kinase family protein |
| AT4G02360 |  | Protein of unknown function, DUF538 |
| AT1G49530 | GGPS6 | Geranylgeranyl pyrophosphate synthase 6 |
| AT1G44350 | ILL6 | IAA-leucine resistant (ILR)-like gene 6 |
| AT2G32510 | MAPKKK17 | Mitogen-activated protein kinase kinase kinase 17 |
| AT1G75230 |  | DNA glycosylase superfamily protein |
| AT1G74950 | JAZ2,TIFY10B | TIFY domain/Divergent CCT motif family protein |

| **Table S11.** Genes encoding proteins that directly interact with RGL3. | | | |
| --- | --- | --- | --- |
| **Gene** | **Alias** | **Annotation** | **Source** |
| AT5G59980 | AtRPP30 | Polymerase/histidinol phosphatase-like | **BioGRID** |
| AT5G59220 | SAG113 | Highly ABA-induced PP2C gene 1 | **BioGRID** |
| AT5G54230 | MYB49 | Myb domain protein 49 | **BioGRID** |
| AT5G48430 |  | Eukaryotic aspartyl protease family protein | **BioGRID** |
| AT5G26280 |  | TRAF-like family protein | **BioGRID** |
| AT1G21700 | CHB4 | SWITCH/sucrose nonfermenting 3C | **BioGRID** |
| AT3G47620 | TCP14 | TEOSINTE BRANCHED, cycloidea and PCF (TCP) 14 | **BioGRID** |
| AT5G14760 | FIN4 | L-aspartate oxidase | **BioGRID** |
| AT5G01820 | ATSR1 | Serine/threonine protein kinase 1 | **BioGRID** |
| AT4G35770 | SEN1 | Rhodanese/Cell cycle control phosphatase superfamily protein | **BioGRID** |
| AT4G24960 | HVA22D | HVA22 homologue D | **BioGRID** |
| AT4G23050 |  | PAS domain-containing protein tyrosine kinase family protein | **BioGRID** |
| AT4G18430 | RABA1e | RAB GTPase homolog A1E | **BioGRID** |
| AT3G48510 |  |  | **BioGRID** |
| AT3G02140 | AFP4 | AFP2 (ABI five-binding protein 2) family protein | **BioGRID** |
| AT2G30360 | PKS5 | SOS3-interacting protein 4 | **BioGRID** |
| AT2G11810 | MGD3 | Monogalactosyldiacylglycerol synthase type C | **BioGRID** |
| AT1G53170 | ERF8 | Ethylene response factor 8 | **BioGRID** |
| AT1G10585 |  | Basic helix-loop-helix (bHLH) DNA-binding superfamily protein | **BioGRID** |
| AT5G66730 | ENY | C2H2-like zinc finger protein | **BioGRID** |
| AT5G27320 | GID1C | Alpha/beta-Hydrolases superfamily protein | **BAR,PAIR** |
| AT3G63010 | GID1B | Alpha/beta-Hydrolases superfamily protein | **BAR,PAIR** |
| AT3G05120 | GID1A | Alpha/beta-Hydrolases superfamily protein | **BAR,PAIR** |
| AT1G75080 | BZR1 | Brassinosteroid signalling positive regulator (BZR1) family protein | **BioGRID** |
| AT1G32640 | ATMYC2 | Basic helix-loop-helix (bHLH) DNA-binding family protein | **BioGRID** |
| AT1G09530 | PIF3 | Phytochrome interacting factor 3 | **BAR,PAIR,BioGRID** |

|  | **Table S12.** GSEA analysis results of RGL3 co-expressed genes. | | | | | | | |
| --- | --- | --- | --- | --- | --- | --- | --- | --- |
|  | Gene Set Name | | | Description | | | FDR | |
|  | MANDAOKAR_JASMONATE_2H_STAMEN_DIFF | | Jasmonate-esponsive genes (2hrs)in stamens of Arabaidopsis thaliana | | | | 2.42E-06 | |
|  | SUGIO_HEAT-SHOCK_DN | | Down-regulated by heat-shock | | | | 0.00024 | |
|  | RESPONSE_TO_JASMONIC_ACID_STIMULUS | | GO:0009753 response to jasmonic acid stimulus | | | | 0.000604 | |
|  | TIFY_FAMILY | | Tify family | | | | 0.00114 | |
|  | RESPONSE_TO_WOUNDING | | GO:0009611 response to wounding | | | | 0.00145 | |
|  | JASMONIC_ACID_METABOLIC_PROCESS | | GO:0009694 jasmonic acid metabolic process | | | | 0.00145 | |
|  | OXYLIPIN_METABOLIC_PROCESS | | GO:0031407 oxylipin metabolic process | | | | 0.00145 | |
|  | SPCH | | SPCH | | | | 0.0141 | |
|  | PIF4 | | LBL | | | | 0.0141 | |
|  | IAA_BIOSYNTHESIS_II | | IAA biosynthesis II | | | | 0.0181 | |
|  | NONAPRENYL_DIPHOSPHATE_BIOSYNTHESIS_III | | Nonaprenyl diphosphate biosynthesis III | | | | 0.0181 | |
|  | GERANYLGERANYLDIPHOSPHATE_BIOSYNTHESIS | | Geranylgeranyldiphosphate biosynthesis | | | | 0.0181 | |
|  | CYTOCHROME_P450_CYP94B | | Cytochrome P450 ,CYP94B | | | | 0.0194 | |
|  | JASMONIC_ACID_BIOSYNTHETIC_PROCESS | | GO:0009695 jasmonic acid biosynthetic process | | | | 0.0249 | |
|  | OXYLIPIN_BIOSYNTHETIC_PROCESS | | GO:0031408 oxylipin biosynthetic process | | | | 0.0249 | |
|  | ACYL_LIPID_METABOLISM_FAMILY_FATTY_ACID_OMEGA_HYDROXYLASE | | Acyl Lipid Metabolism Family ,Fatty Acid omega Hydroxylase | | | | 0.029 | |
|  | PRR5_inhouse_predicted | | PRR5_inhouse_predicted_TSS3kb+genebody+TTS1kb | | | | 0.0496 | |
| **Table S13.** RGL3 co-expressed gene promoter region cis-acting element analysis. | | | | | | | |  |
| **Motif name** | | **Sequence** | | | **P-value** | **description** | |  |
| CE3OSOSEM | | AACGCGTGTC | | | 0 | CE3 (Coupling Element 3)" found in the promoter of the rice Osem gene; Required for ABA-responsiveness and VP1 activation; Binding site of TRAB1; | |  |
| ABRE3HVA1 | | GCAACGTGTC | | | 1.00E-06 | ABA responsive element, ABRE3, found in barley (H.v.) HVA1 gene encoding a class 3 late embryogenesis-abundant protein; | |  |
| JASE1ATOPR1 | | CGTCAATGAA | | | 2.78E-03 | Involved in up-regulation by both senescence and JA; | |  |
| OBP1ATGST6 | | TACACTTTTGG | | | 6.03E-03 | Characterization of salicylic acid-responsive; | |  |
| PROXBBNNAPA | | CAAACACC | | | 3.40E-02 | Required for seed specific expression and ABA responsiveness; | |  |
| MYB2CONSENSUSAT | | [TC]AAC[GT]G | | | 4.04E-02 | MYB recognition site found in the promoters of the dehydration-responsive gene rd22 and many other genes in Arabidopsis; | |  |
| MYB1AT | | [AT]AACCA | | | 4.47E-02 | MYB recognition site found in the promoters of the dehydration-responsive gene rd22 and many other genes in Arabidopsis; | |  |
| QARBNEXTA | | AACGTGT | | | 3.13E-04 | In response to wounding and tensile stress | |  |
| HSE | | CT[ATCG]GAA[ATCG]{2}TTC[ATCG]AG | | | 7.91E-04 | HSE (heat shock response element) | |  |
| MYB2AT | | TAACTG | | | 6.46E-03 | Rresponsive to water stress in Arabidopsis; An Arabidopsis myb homolog is induced by dehydration stress. | |  |
| ABRELATERD1 | | ACGTG | | | 1.21E-02 | Induction by dehydration stress and dark-induced senescence. | |  |
| ANAC019 | | CACG | | | 3.09E-02 | A drought-responsive cis-element in the early responsive to dehydration stress. | |  |
| ACGTATERD1 | | ACGT | | | 3.14E-02 | Induction by dehydration stress and dark-induced senescence. | |  |

| **Table S14.** GSEA analysis results of RGL3 interacting proteins. | | |
| --- | --- | --- |
| **Gene Set Name** | **Description** | **FDR** |
| RESPONSE_TO_ABSCISIC_ACID_STIMULUS | GO:0009737 response to abscisic acid stimulus | 3.10E-08 |
| REGULATION_OF_GIBBERELLIC_ACID_MEDIATED_SIGNALING_PATHWAY | GO:0009937 regulation of gibberellic acid mediated signaling pathway | 5.78E-07 |
| GIBBERELLIC_ACID_MEDIATED_SIGNALING_PATHWAY | GO:0009740 gibberellic acid mediated signaling pathway | 2.07E-06 |
| POSITIVE_REGULATION_OF_GIBBERELLIC_ACID_MEDIATED_SIGNALING_PATHWAY | GO:0009939 positive regulation of gibberellic acid mediated signaling pathway | 6.95E-06 |
| RESPONSE_TO_JASMONIC_ACID_STIMULUS | GO:0009753 response to jasmonic acid stimulus, GOslim:biological_process | 1.96E-05 |
| ABSCISIC_ACID_MEDIATED_SIGNALING_PATHWAY | GO:0009738 abscisic acid mediated signaling pathway | 1.84E-04 |
| CELLULAR_RESPONSE_TO_ABSCISIC_ACID_STIMULUS | GO:0071215 cellular response to abscisic acid stimulus | 2.34E-04 |
| RESPONSE_TO_SALT_STRESS | GO:0009651 response to salt stress | 2.63E-04 |
| RESPONSE_TO_OSMOTIC_STRESS | GO:0006970 response to osmotic stress | 3.85E-04 |
| DIJK_DEHYDRATION-STRESS_UP | Up-Further analysis indicated 640 genes increased, their transcript levels by 4-fold or more (p <= 0.001) as result of the dehydration stress. | 9.33E-04 |
| RESPONSE_TO_SALICYLIC_ACID_STIMULUS | GO:0009751 response to salicylic acid stimulus | 2.64E-03 |
| JASMONIC_ACID_MEDIATED_SIGNALING_PATHWAY | GO:0009867 jasmonic acid mediated signaling pathway | 4.88E-03 |
| CELLULAR_RESPONSE_TO_JASMONIC_ACID_STIMULUS | GO:0071395 cellular response to jasmonic acid stimulus | 4.88E-03 |
| SALICYLIC_ACID_MEDIATED_SIGNALING_PATHWAY | GO:0009863 salicylic acid mediated signaling pathway | 0.0104 |
| CELLULAR_RESPONSE_TO_SALICYLIC_ACID_STIMULUS | GO:0071446 cellular response to salicylic acid stimulus | 0.0105 |
| NEGATIVE_REGULATION_OF_ABSCISIC_ACID_MEDIATED_SIGNALING_PATHWAY | GO:0009788 negative regulation of abscisic acid mediated signaling pathway | 0.0153 |
| REGULATION_OF_ABSCISIC_ACID_MEDIATED_SIGNALING_PATHWAY | GO:0009787 regulation of abscisic acid mediated signaling pathway | 0.0345 |
| REGULATION_OF_TRANSCRIPTION_FROM_RNA_POLYMERASE_II_PROMOTER_IN_RESPONSE_TO_OXIDATIVE_STRESS | GO:0043619 regulation of transcription from RNA polymerase II promoter in response to oxidative stress | 0.0482 |
|  |  |  |

**Table S15.** GSEA analysis results of ERF4 co-expressed genes.

| **Gene Set Name** | **Description** | **FDR** |
| --- | --- | --- |
| NEGATIVE_REGULATION_OF_ETHYLENE_MEDIATED_SIGNALING_PATHWAY | GO:0010105 negative regulation of ethylene mediated signaling pathway | 5.83E-04 |
| NEGATIVE_REGULATION_OF_TWO-COMPONENT_SIGNAL_TRANSDUCTION_SYSTEM_(PHOSPHORELAY) | GO:0070298 negative regulation of two-component signal transduction system (phosphorelay) | 5.83E-04 |
| REGULATION_OF_ETHYLENE_MEDIATED_SIGNALING_PATHWAY | GO:0010104 regulation of ethylene mediated signaling pathway | 6.90E-04 |
| REGULATION_OF_TWO-COMPONENT_SIGNAL_TRANSDUCTION_SYSTEM_(PHOSPHORELAY) | GO:0070297 regulation of two-component signal transduction system (phosphorelay) | 6.90E-04 |
| NEGATIVE_REGULATION_OF_CELL_COMMUNICATION | GO:0010648 negative regulation of cell communication | 3.89E-03 |
| NEGATIVE_REGULATION_OF_SIGNALING | GO:0023057 negative regulation of signaling | 3.89E-03 |
| NEGATIVE_REGULATION_OF_SIGNAL_TRANSDUCTION | GO:0009968 negative regulation of signal transduction | 3.89E-03 |
| RESPIRATORY_BURST | GO:0045730 respiratory burst, GOslim:biological_process | 1.22E-02 |
| RESPIRATORY_BURST_INVOLVED_IN_DEFENSE_RESPONSE | GO:0002679 respiratory burst involved in defense response | 1.22E-02 |
| ETHYLENE_MEDIATED_SIGNALING_PATHWAY | GO:0009873 ethylene mediated signaling pathway, GOslim:biological_process | 6.65E-06 |
| CELLULAR_RESPONSE_TO_ETHYLENE_STIMULUS | GO:0071369 cellular response to ethylene stimulus | 6.65E-06 |
| REGULATION_OF_SIGNALING | GO:0023051 regulation of signaling, GOslim:biological_process | 2.02E-02 |
| REGULATION_OF_SIGNAL_TRANSDUCTION | GO:0009966 regulation of signal transduction | 2.02E-02 |
| TWO-COMPONENT_SIGNAL_TRANSDUCTION_SYSTEM_(PHOSPHORELAY) | GO:0000160 two-component signal transduction system (phosphorelay) | 9.71E-06 |
| REGULATION_OF_CELL_COMMUNICATION | GO:0010646 regulation of cell communication | 2.04E-02 |
| INTRACELLULAR_SIGNAL_TRANSDUCTION | GO:0035556 intracellular signal transduction | 2.94E-02 |
| IMMUNE_EFFECTOR_PROCESS | GO:0002252 immune effector process | 4.05E-02 |
| RESPONSE_TO_ETHYLENE_STIMULUS | GO:0009723 response to ethylene stimulus | 9.85E-05 |
| RESPONSE_TO_CHITIN | GO:0010200 response to chitin | 4.19E-03 |
| HORMONE-MEDIATED_SIGNALING_PATHWAY | GO:0009755 hormone-mediated signaling pathway | 5.83E-04 |
| RESPONSE_TO_ABSCISIC_ACID_STIMULUS | GO:0009737 response to abscisic acid stimulus | 9.39E-03 |
| CELLULAR_RESPONSE_TO_HORMONE_STIMULUS | GO:0032870 cellular response to hormone stimulus | 5.83E-04 |
| CELLULAR_RESPONSE_TO_ENDOGENOUS_STIMULUS | GO:0071495 cellular response to endogenous stimulus | 9.96E-04 |
| RESPONSE_TO_CARBOHYDRATE_STIMULUS | GO:0009743 response to carbohydrate stimulus | 1.83E-02 |
| CELLULAR_RESPONSE_TO_ORGANIC_SUBSTANCE | GO:0071310 cellular response to organic substance | 3.59E-03 |
| RESPONSE_TO_HORMONE_STIMULUS | GO:0009725 response to hormone stimulus | 4.66E-03 |
| CELLULAR_RESPONSE_TO_CHEMICAL_STIMULUS | GO:0070887 cellular response to chemical stimulus | 4.75E-03 |
| RESPONSE_TO_ENDOGENOUS_STIMULUS | GO:0009719 response to endogenous stimulus | 7.18E-03 |
| SIGNAL_TRANSDUCTION | GO:0007165 signal transduction | 7.85E-03 |
| SIGNAL_TRANSMISSION | GO:0023060 signal transmission | 9.33E-03 |
| CELLULAR_RESPONSE_TO_STIMULUS | GO:0051716 cellular response to stimulus | 2.04E-02 |
| SIGNALING | GO:0023052 signaling | 2.04E-02 |
| RESPONSE_TO_ORGANIC_SUBSTANCE | GO:0010033 response to organic substance | 3.51E-03 |
| RESPONSE_TO_CHEMICAL_STIMULUS | GO:0042221 response to chemical stimulus | 1.22E-02 |

## Supplementary Figures


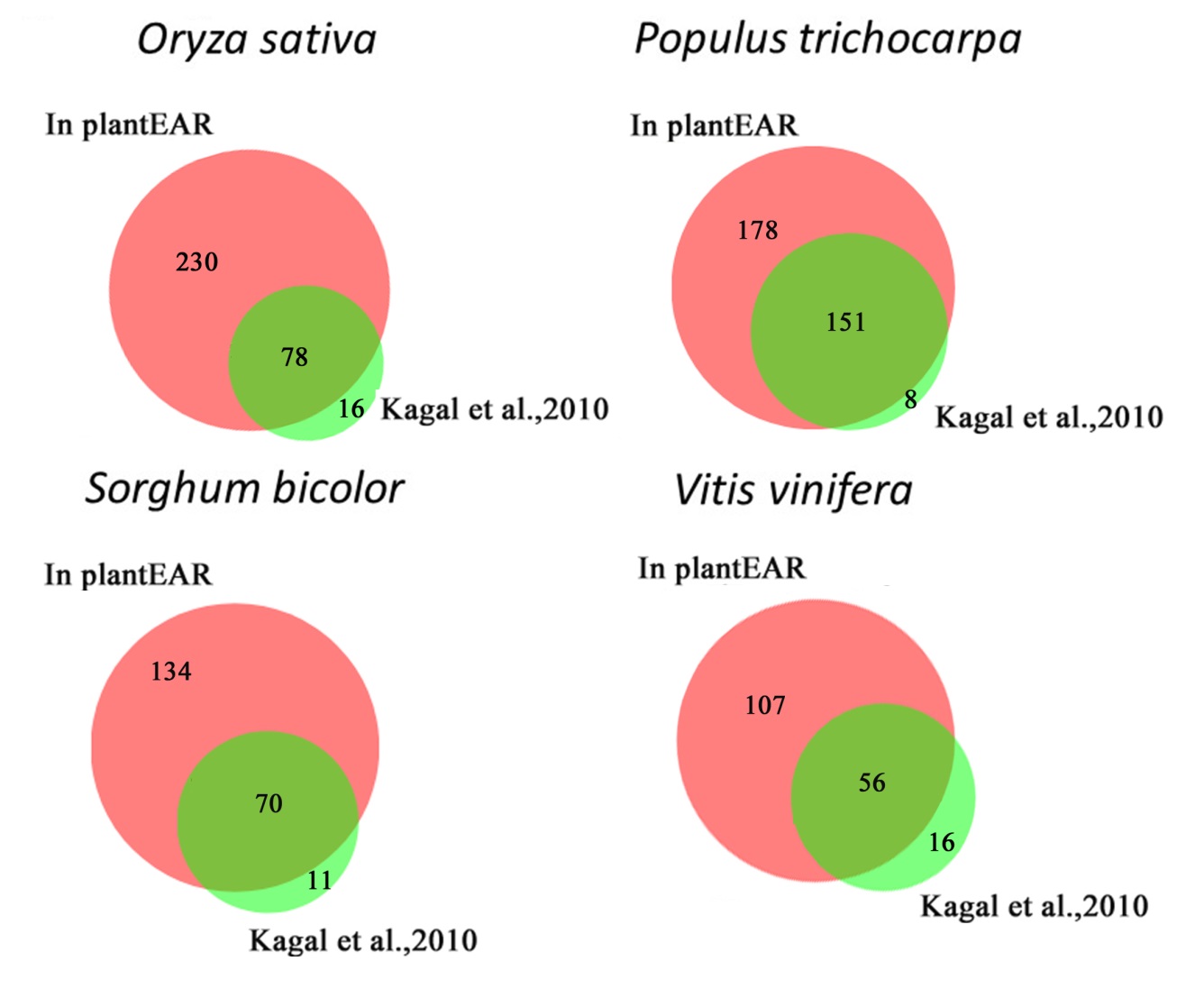


**Figure S1.** The comparison of the results of predicted EAR motif-containing proteins in four species (*Oryza sativa*, *Populus trichocarpa*, *Sorghum bicolor*, *Vitis vinifera*) between previous literature and plantEAR.


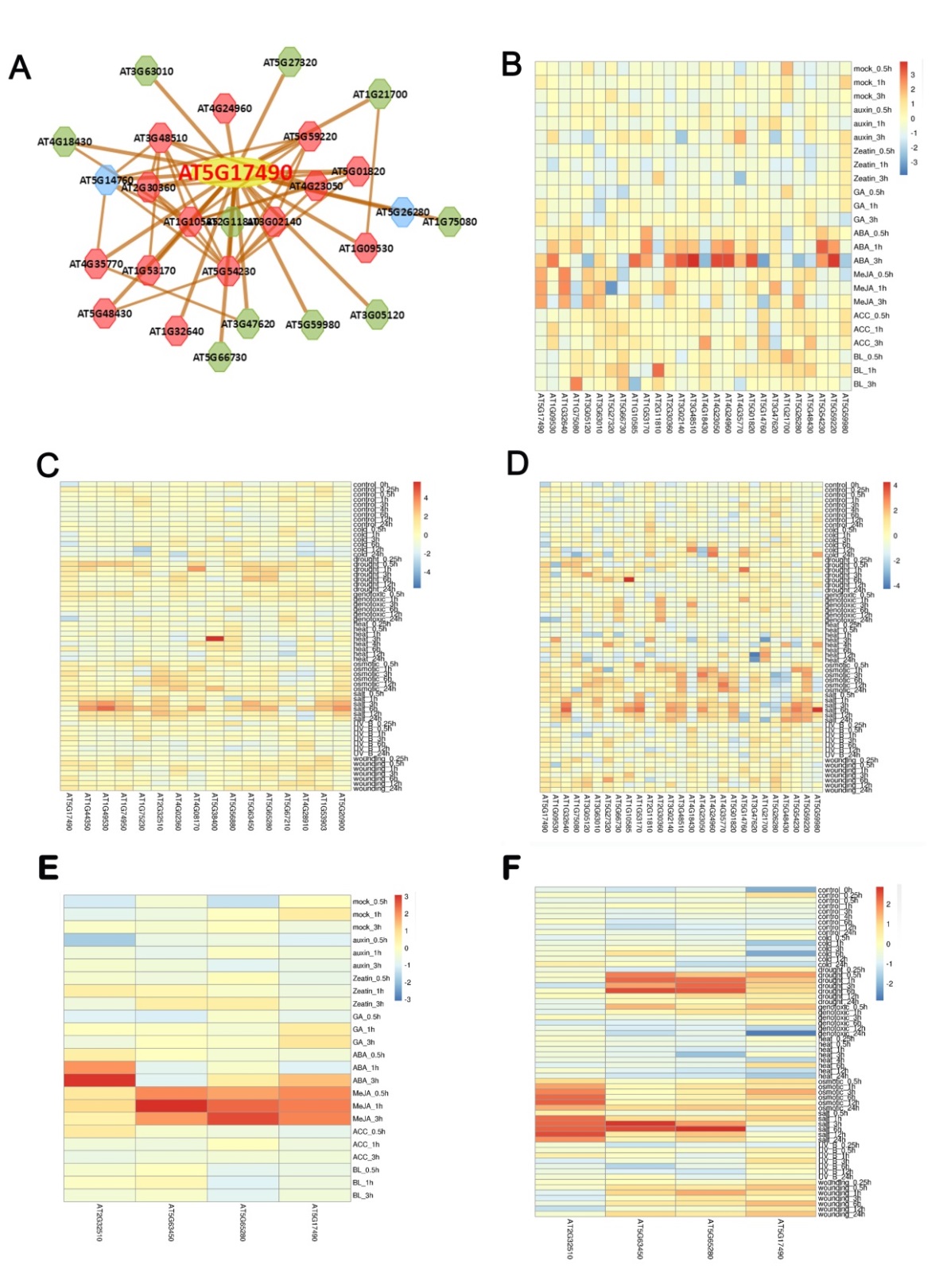


**Figure S2.** PPI and co-expression network of RGL3 with the expression view in *Arabidopsis*. (A) PPI network of RGL3 with the expression view under salt stress for 3 h. (B) Heatmap of RGL3 interaction protein-coding genes under different hormone treatments. (C) Heatmap analysis of RGL3 co-expressed genes under different stress treatments. (D) Heatmap of the RGL3 interaction protein-coding genes under stress treatment. (E) Expression view of the module containing RGL3 under different hormone treatments. (F) Expression view of the module containing RGL3 under different stresses.

**
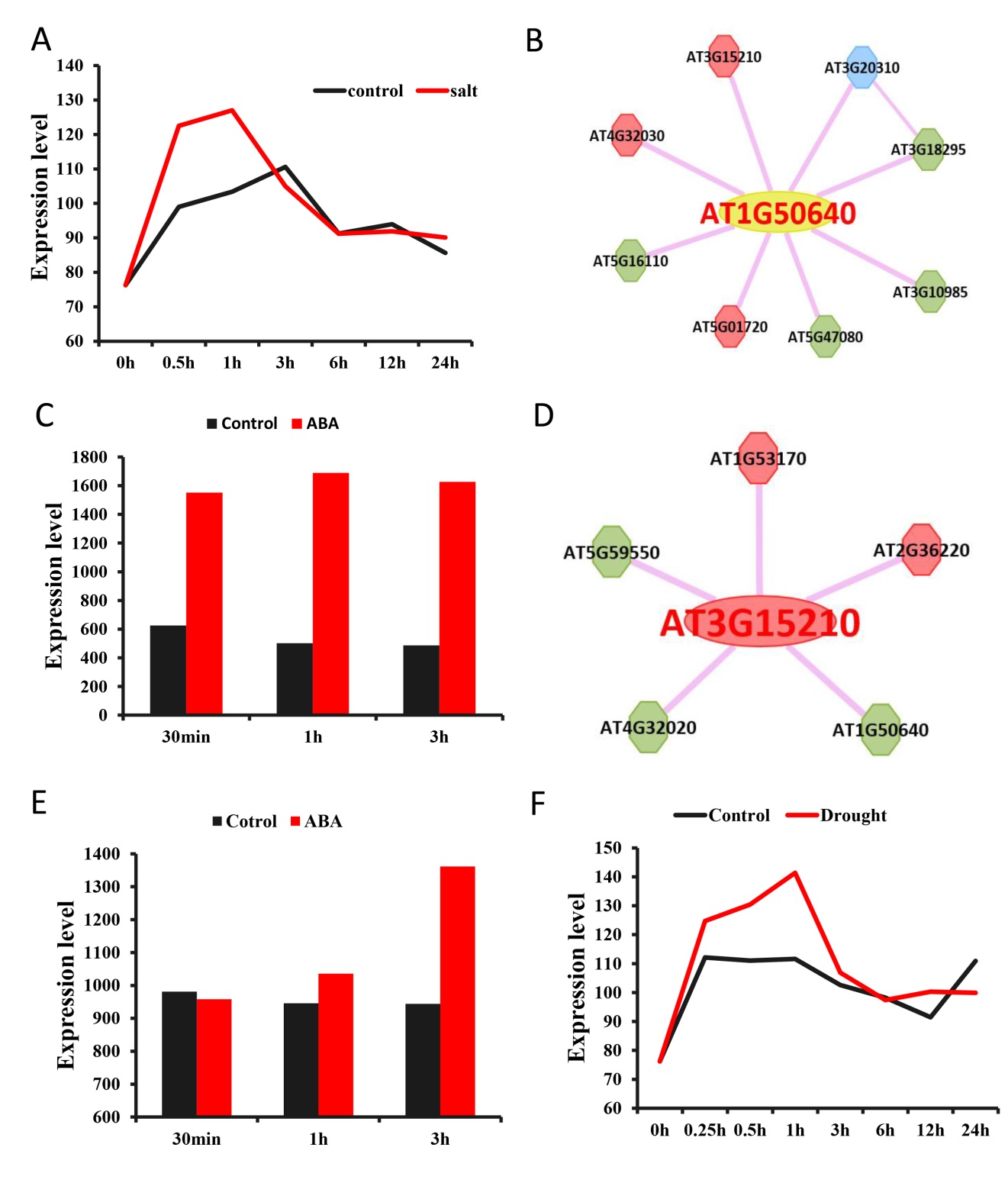
**

**Figure S3.** Functional analysis of ERF3, ERF4 and ERF5 using plantEAR. (A) Expression levels of ERF3 under salt stress at different time points. (B) Changes in ERF3 co-expressed genes expression levels under 6 h of salt stress. (C) Expression of ERF4 under ABA treatment at different time points. (D) Changes in ERF4 co-expressed gene expression levels under ABA treatment at 0.5 h. (E) Expression of ERF7 under ABA treatment at different time points. (F) Expression of ERF7 under drought stress at different time points.


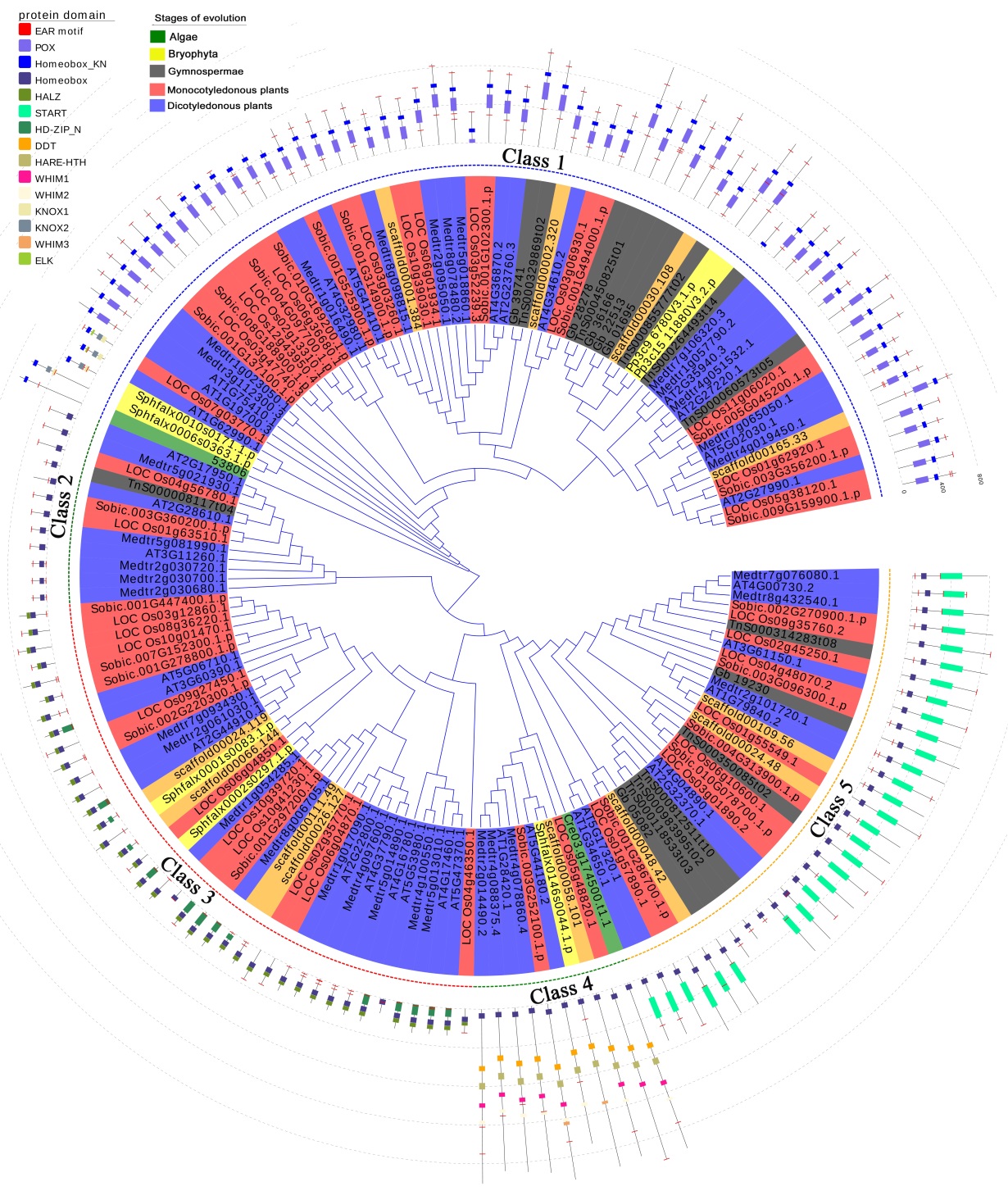


**Figure S4**. Phylogenetic tree of EAR motif-containing proteins in the HB family across different species. The unrooted tree was divided into five classes. As shown on the top left, proteins with different background colours represent proteins in different evolutionary stages. The outermost is the protein structure, and the different colours represent different domains.


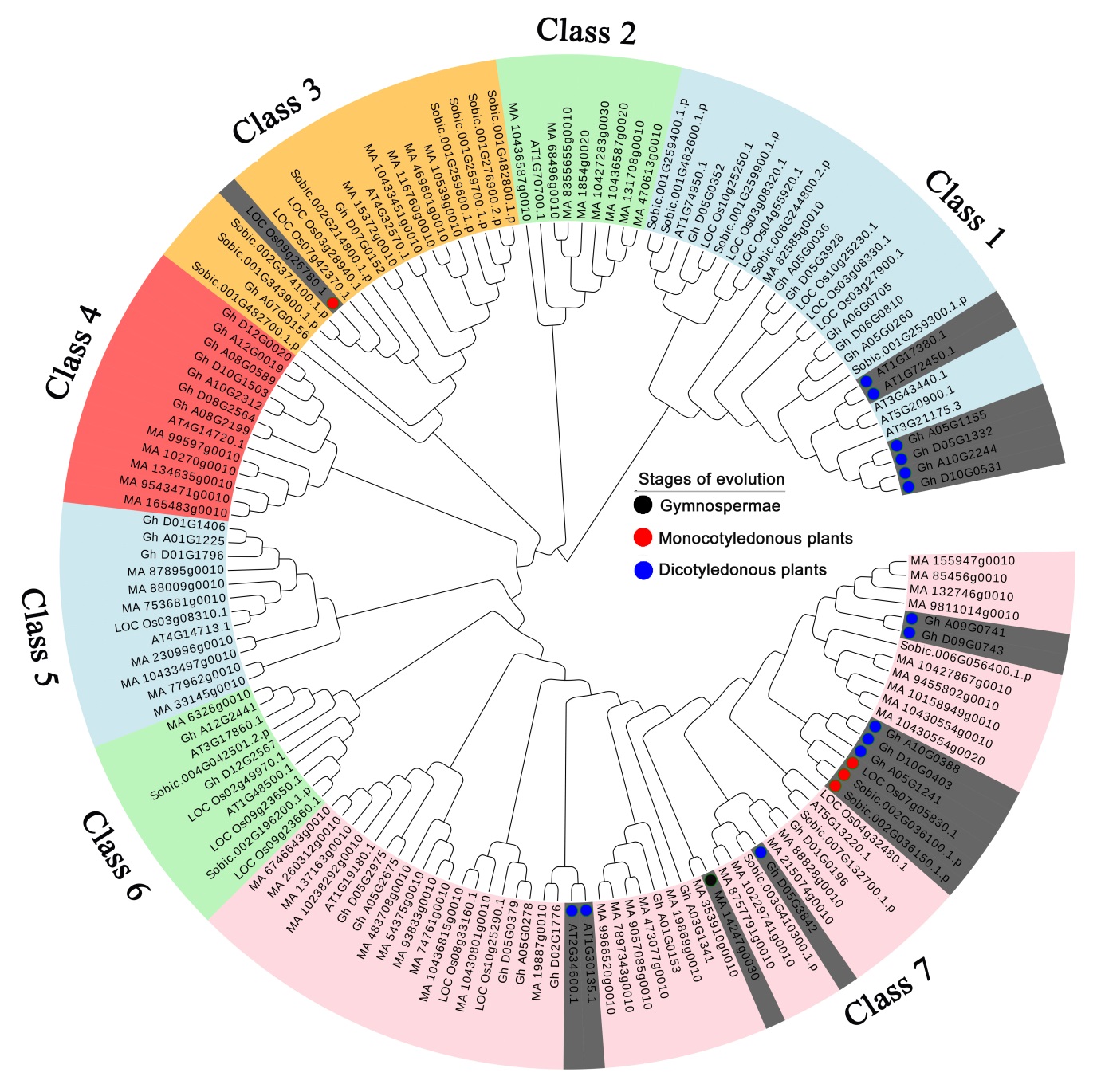


**Figure S5.** Phylogenetic tree of EAR motif-containing proteins in Tify family across different species. The unrooted tree was divided into seven classes. The black circle represents the EAR motif-containing protein in gymnosperms. Blue circles represent the EAR motif-containing proteins in dicotyledonous plants. Red circles represent the EAR motif-containing proteins in monocotyledonous plants.
